# Modulation of AMPK by esomeprazole and canagliflozin mitigates methotrexate-induced hepatotoxicity: involvement of MAPK/JNK/ERK, JAK1/STAT3, and PI3K/Akt signaling pathways

**DOI:** 10.1007/s00210-025-03908-3

**Published:** 2025-03-07

**Authors:** Ahmed M. El-Dessouki, Mohamed E. Kaml, Mohammed F. EL-Yamany

**Affiliations:** 1 Pharmacology and Toxicology Department, Faculty of Pharmacy, Ahram Canadian University (ACU), 6th of October City, Giza, 12566 Egypt; 2 Pharmacology and Toxicology Department, Faculty of Pharmacy, Cairo University, Giza, 11562 Egypt

**Keywords:** Methotrexate, Esomeprazole, Canagliflozin, Hepatoxicity, AMPK, MAPK

## Abstract

This research investigated the hepatoprotective effects of esomeprazole (ESOM) and canagliflozin (CANA) against methotrexate-induced liver toxicity, focusing on AMPK modulation and its regulation of MAPK/JNK/ERK, JAK1/STAT3, and PI3K/Akt pathways. Fifty male Wistar rats were divided into five groups: control, MTX, and three pretreatment groups receiving ESOM (30 mg/kg), CANA (30 mg/kg), or their combination. ESOM and CANA were administered for 8 days before and 1 day after a single MTX injection (20 mg/kg, intraperitoneally) on day 9 to induce hepatotoxicity. Liver injury, oxidative stress, inflammation, and apoptosis were assessed using biochemical, histopathological, immunohistochemical, qRT-PCR, and western blot analyses. Data were analyzed by one-way analysis of variance (ANOVA) and Tukey’s post hoc test, with significance at *p* < 0.05. Results were presented as mean ± standard error (SE). Rats that received MTX showed significant liver damage, marked by elevated ALT, AST, MDA, MPO, iNOS, TNF-α, IL-6, and IL-1β levels (*p* < 0.01) and decreased antioxidant enzymes (HO-1, Nrf2, and GSH). Immunohistochemistry revealed increased NF-kB p65 and caspase-9 expression (*p* < 0.01), correlating with histopathological changes. Pretreatment with ESOM and CANA reduced liver enzyme levels, improved histology, restored antioxidant balance, and inhibited inflammatory pathways via p38MAPK/NF-kB p65 and JAK1/STAT3 (*p* < 0.01). Moreover, ESOM and CANA preserved PI3K/Akt activity and prevented caspase-dependent apoptosis (*p* < 0.01). Additionally, the combination treatment showed synergistic hepatoprotective effects, demonstrated by significant improvements in all measured parameters. These findings suggested that ESOM and CANA had significant potential as therapeutic agents for alleviating MTX-induced hepatotoxicity and warranted further investigation in future research.

**Supplementary Information:**

The online version contains supplementary material available at 10.1007/s00210-025-03908-3.

## Introduction

Drug-induced liver injury (DILI) is a pathological condition characterized by structural and functional liver abnormalities caused by medicinal compounds. The liver’s central role in detoxification renders it vulnerable to drug toxicity (Li et al. 2022c). Hepatotoxicity often leads to the market withdrawal of drugs once their tendency to induce DILI is discovered during clinical use (Hosack et al. 2023). Drug metabolism can generate reactive metabolites, increasing reactive oxygen species (ROS), oxidative damage, and cell death. While hepatocytes have antioxidant defenses, their dysfunction can trigger DILI. ROS-induced inflammation and immune responses further exacerbate the condition (Villanueva-Paz et al. 2021).

Methotrexate (MTX), an anti-metabolite and chemotherapeutic agent, is widely used for malignancies and autoimmune diseases like psoriasis, rheumatoid arthritis, and inflammatory bowel disease (AlAmeel et al. 2022; El-Dessouki et al. 2024b). The administration of MTX is linked to an enhanced generation of ROS, which cause oxidative damage to cellular components and disrupt redox homeostasis (Schmidt et al. 2022b). The resulting oxidative stress activates pro-inflammatory signaling pathways, such as the MAPK/NF-κB axis, leading to increased expression of cytokines like TNF-α, IL-1β, and IL-6 (Abdelall et al. 2024). Additionally, MTX has been shown to impair antioxidant defense systems, including the Nrf2/HO-1 pathway, further exacerbating liver injury (Mahmoud et al. 2020). This is coupled with the activation of apoptotic pathways, such as the JAK1/STAT3 and PI3K/Akt cascades, which promote mitochondrial dysfunction and cell death (Ebrahimi et al. 2019). Together, these processes highlight the complex interplay of oxidative stress, inflammation, and apoptosis in the pathogenesis of MTX-induced liver damage.

AMP-activated protein kinase (AMPK) serves as a vital energy sensor and regulator of cellular metabolic processes, playing a significant role in maintaining energy homeostasis (Townsend and Steinberg 2023). Notably, AMPK activation has been shown to improve insulin sensitivity, reduce inflammation, and promote longevity (Ge et al. 2022). Additionally, it plays a significant role in regulating mitochondrial dynamics, essential for cellular adaptation to metabolic stress. As a result, AMPK not only maintains cellular energy levels but also contributes to cellular resilience and adaptation (Aslam and Ladilov 2022). Given its broad regulatory impact, AMPK is increasingly recognized as a therapeutic target for a range of diseases, including metabolic disorders, cardiovascular diseases, and cancer (Bu et al. 2022; Keerthana et al. 2023). Therefore, activating AMPK is viewed as a potential strategy to mitigate the hepatotoxic effects caused by MTX.

Esomeprazole (ESOM), a benzimidazole derivative, is a well-known proton pump inhibitor (PPI) used in the treatment of gastrointestinal conditions by inactivating H+/K+ ATPase (Xu et al. 2020). PPIs function by inhibiting hydrogen potassium adenosine triphosphatase, thereby decreasing gastric acid secretion from parietal cells. Recently, PPIs have demonstrated additional pharmacological effects, such as anti-inflammatory, antitumor, and antioxidant activities (Liu et al. 2022). Furthermore, recent findings indicate that PPIs may offer therapeutic advantages for fibrotic conditions like thyroid eye disease and interstitial lung disease (Hammond et al. 2019). PPIs have also been shown to protect against mortality induced by lipopolysaccharides in a murine model of fatal endotoxic shock (Li et al. 2020).

Canagliflozin, a sodium-glucose co-transporter 2 (SGLT2) inhibitor, reduces glucose reabsorption in renal tubules, thereby promoting its excretion through urine (Yang et al. 2021). Research shows canagliflozin has antioxidant and anti-inflammatory properties, reducing heart and kidney remodeling, arterial stiffness, and blood pressure (Hasan et al. 2020). Animal studies reveal it lowers hepatic lipids, protecting against nonalcoholic fatty liver disease in diabetic rats (Xu et al. 2022). Additionally, it reduces liver fat, triglycerides, and glycogen, mitigating hepatic steatosis (Kabil and Mahmoud 2018).

Currently, no studies have specifically explored the protective effects of ESOM and CANA against MTX-induced hepatotoxicity. Therefore, this research aims to investigate the hepatoprotective effects of esomeprazole and canagliflozin against methotrexate-induced liver toxicity, focusing on AMPK modulation and its regulation of MAPK/JNK/ERK, JAK1/STAT3, and PI3K/Akt pathways, while acknowledging limitations in dose-response analysis and impact on methotrexate’s anticancer efficacy.

## Materials and methods

### Drugs and chemicals

Methotrexate, provided in a vial containing 50 mg/2 mL, was sourced from Mylan N.V., located in West Virginia, USA, while ESOM and CANA were supplied by Eva Pharma for Pharmaceutical and Medical Appliance Company (Cairo, Egypt) and Janssen Pharmaceutical Company (Inc., UK), respectively. Both ESOM and CANA were prepared for oral administration by dissolving them in 1% Tween 80. The formalin solution used in the experiments was sourced from Merck Company, Germany. Specific assay kits, detailed for each biochemical parameter under investigation, were used for biochemical evaluations. All other chemicals required for the experiments were provided by Sigma-Aldrich, Munich, Germany.

### Animals

Adult male albino rats, each initially weighing about 200 ± 20 g, were sourced from the Cairo University animal house in Cairo, Egypt. Before beginning the experiments, the rats were acclimated for a week in a controlled environment, maintaining a temperature of 25 °C ± 0.5 and humidity at 55% ± 1%, with a 12-h light/dark cycle. They were given a regular diet and had access to water. Animal handling and care followed the guidelines established by the Faculty of Pharmacy Research Ethics Committee at Cairo University, Egypt (Ethics code: PT 3083), consistent with the “Principles of Laboratory Animal Care” (NIH publication No. 85-23, revised 1985).

### Experimental design

**Fig. 1:**
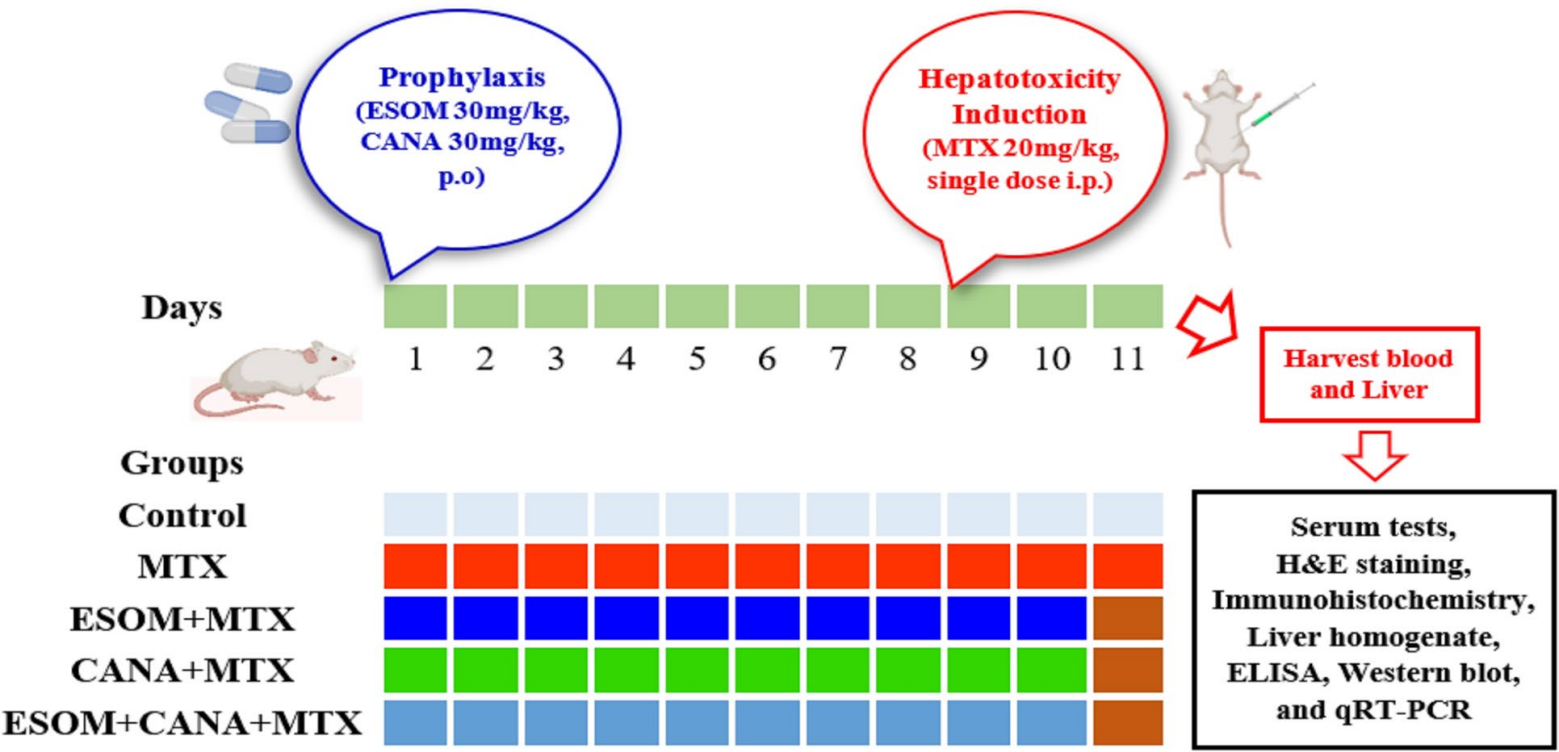
The experimental design is illustrated in the schematic diagram. Rats were separated into five groups. The control group was given 1% Tween 80, whereas the MTX group received methotrexate at a dose of 20 mg/kg. Pretreatment groups received ESOM (30 mg/kg), CANA (20 mg/kg), or both, starting 8 days before and continuing 1 day after MTX. Rats were sacrificed for biochemical, histopathological, immunohistochemical, qRT-PCR, and western blot analyses

As shown in Figure 1, 50 male Wistar rats were separated into five groups of 10 rats each. The control group was given 1% Tween 80. To induce hepatotoxicity, the MTX group was administered a single intraperitoneal injection of MTX at a dose of 20 mg/kg on the ninth day (Mehrzadi et al. 2018). Three pretreatment groups were given ESOM at 30 mg/kg (Eltahir and Nazmy 2018), CANA at 30 mg/kg (Morsy et al. 2021), or a combination of ESOM (30 mg/kg) and CANA (30 mg/kg), starting 8 days prior to MTX administration and continuing for 1 day. On the 11th day, the rats were sacrificed to collect blood samples for serum separation and liver tissues for biochemical, histopathological, immunohistochemical, qRT-PCR, and western blot analyses.

### Sample collection and preparation

Animals used for experimentation were administered thiopental sodium for anesthesia at a dose of 50 mg/kg prior to being euthanized. Blood samples were drawn from the retro-orbital plexus to collect serum, which was then centrifuged at 4000 rpm for 10 min. The obtained serum was placed in sterile tubes and stored at − 20 °C for later assessment of alanine transaminase (ALT) and aspartate aminotransferase (AST) activities. The liver tissues were subsequently removed, rinsed with saline, and divided into four sections: the first two segments were quickly frozen in liquid nitrogen for future qRT-PCR and western blot assays; for histopathological and immunohistochemical analyses, the third section was preserved in 10% buffered formalin; the fourth segment was processed using an Omni-125 handheld homogenizer in a 10% (wt./vol) ice-cold solution of 0.01 M phosphate-buffered saline (pH 7.4). After centrifugation, the supernatant was collected and preserved at − 80 °C for later ELISA analysis.

### Evaluation of liver serum biomarkers

The activities of liver enzymes, particularly AST and ALT, in rat serum were measured using ELISA kits sourced from Biomatik Co. (Cat. Nos. EKE62019 and EKU02211, respectively, Delaware, USA) following the manufacturer’s procedures.

### Evaluation of oxidative stress parameters

Oxidative stress markers, including iNOS, MDA, and MPO, were quantified alongside antioxidant capacity markers such as GSH and Nrf2 levels in the liver. These evaluations utilized ELISA kits sourced from various suppliers: Cusabio for iNOS (Cat. No. CSB-E08325r, Houston, USA), MyBioSource for MDA (Cat. No. MBS268427, San Diego, USA), BioVision for MPO (Cat. No. E4581-100, Milpitas, USA), Cloud-Clone Corp. for GSH (Cat. No. CEA294Ge, Katy, USA), and BT-Lab for Nrf2 (Cat. No. E1083Ra, Shanghai, China), following each manufacturer’s procedures (Hefny et al. 2024).

### Measurement of pro-inflammatory cytokines

The concentrations of pro-inflammatory cytokines, specifically TNF-α and IL-6, in liver tissues were quantitatively assessed using ELISA kits. These kits, obtained from R&D Systems (Cat. No. RTA00-1 for TNF-α, Minneapolis, USA) and Elabscience (Cat. No. EL-R0015 for IL-6, Houston, USA), were employed following the manufacturers’ protocols

### Estimation of apoptotic markers in liver

The quantification of apoptotic markers, including p53 and caspase-3, in liver tissues was conducted using ELISA kits. The kits were sourced from Cusabio for p53 (Cat. No. CSB-E08336r, Houston, USA) and Elabscience for caspase-3 (Cat. No. EL-R0160, Houston, USA), were employed following the manufacturers’ protocols (Tawfik et al. 2024).

### Histopathology and immunohistochemistry (IHC)

Liver samples were immediately placed in a 10% solution of neutral buffered formalin for preservation for 24 h following collection. After fixation, the tissues were rinsed, dehydrated using a series of increasing ethanol concentrations, cleared with xylene, and then embedded in paraffin wax. Using a microtome, longitudinal sections of the paraffin-embedded tissues, each 5 µm thick, were prepared and mounted on glass slides. Following the deparaffinization of sections, they were stained with hematoxylin and eosin and then examined under a light microscope in a blinded manner for histopathological evaluation (El-Gamil et al. 2024).

For the immunohistochemical analysis, liver sections, each 5 μm thick, underwent deparaffinization and were incubated overnight at 4 °C with monoclonal antibodies targeting caspase 9 (ABclonal Co.; Cat. No. A11910) and nuclear factor kappa B p65 (Abcam Co.; Cat. No. ab131100). Following incubation, sections were exposed to horseradish peroxidase-conjugated secondary antibodies from Abcam. Visualization was achieved using a 2% diaminobenzidine solution in 50 mM Tris buffer (pH 7.6). The immunohistochemically stained slides were examined with an Olympus CH2 light microscope. Quantification of the brown-stained regions corresponding to caspase-9 and NF-κB p65 was performed using ImageJ software by analyzing five fields per section, applying a scale bar of 20 microns, and calculating the percentage of positively stained areas (Antar et al. 2024; Shaldam et al. 2024).

### Quantitative real-time polymerase chain reaction (qRT-PCR)

To precisely evaluate the hepatic gene expression levels of AMPK, PI3K, AKT, IL-1β, and HO-1, the technique of quantitative real-time polymerase chain reaction (qRT-PCR) was employed. RNA was extracted from liver tissues using the TRIzol reagent (Thermo Fisher Scientific, USA). The RNA’s concentration and quality were verified with a Nanodrop spectrophotometer (Thermo Fisher Scientific Inc., USA), specifically by measuring absorbance at 260 nm and evaluating the 260/280 nm ratio. Only RNA samples with a purity index of 1.8 or greater were included in the qRT-PCR analysis. For cDNA synthesis, 1 µg of total RNA was reverse transcribed using the Qiagen RT-PCR kit (Qiagen, USA) as per the manufacturer’s protocol. qRT-PCR assays were carried out utilizing the Maxima SYBR Green/Fluorescein qPCR Master Mix from Thermo Scientific (USA) and executed on the Rotor-Gene Q system produced by Qiagen (USA).

The reaction mix included 12.5 µL of SYBR® Green PCR master mix, 2 µL of forward primer, 2 µL of reverse primer, 5.5 µL of RNase/DNase-free water, and 3 µL of cDNA. The mixture was gently vortexed and centrifuged to ensure thorough mixing. Thermal cycling was performed starting with a 10-min DNA polymerase activation at 95 °C. This was succeeded by 45 cycles that involved denaturation at 95 °C for 10 s, annealing at 60 °C for 15 s, and elongation at 72 °C for 15 s. The primers for each gene, detailed in Table 1, were designed using the PrimerQuest Tool, which is based on gene sequences available on PubMed. Gene expression levels were normalized to the reference gene rat beta-actin (β-actin), and relative expression levels were computed using the 2−ΔΔCT method.

**Table 1:** Primers used for qRT-PCR

Gene	Description	Primer sequence	Reference sequence	Product size
AMPK	Forward	5′-CATTCTTGGTTGCCGAAACA-3′	NM_023991.1	70
	Reverse	5′-TGTTTGGATTTCTGTGGGTT-3′		
PI3K	Forward	5′-CCCGGGTAGGTTTGAATTCGT-3′	NM_001371300.2	95
	Reverse	5`- ATGCCCTAGGTGACCTGACA-3′		
Akt	Forward	5′-GAGGAGCGGGAAGAGTG-3′	NM_033230.3	199
	Reverse	5′-TGCCCTTGCCCAGTAG-3′		
IL-1β	Forward	5′-ATCTCACAGCAGCATCTCGACAAG-3′	NM_031512.2	194
	Reverse	5′-CACACTAGCAGGTCGTCATCATCC-3′		
HO-1	Forward	5′-AGAGTTTCTTCGCCAGAGGC-3′	NM_012580.2	265
	Reverse	5′-AGGCCCAAGAAAAGAGAGCC-3′		
β-Actin	Forward	5′-TGGAGAAGAGCTATGAGCTGCCTG-3′	NM_031144.3	202
	Reverse	5′-GTGCCACCAGACAGCACTGTGTTG-3′		

### Western blot

The rat tissue or cell lysate mixture, consisting of RIPA buffer (Thermo Fisher Scientific, 89900, USA), protease inhibitor (Sigma-Aldrich, P8340, USA), and phosphatase inhibitor (Sigma-Aldrich, P8340, USA), was homogenized to extract total protein. The BCA kit (Thermo Fisher Scientific, 23225, USA) quantified the protein samples before boiling them in Laemmli buffer (Bio-Rad, 1610747, USA) at 95 °C for 5 min. Sodium dodecyl sulfate–polyacrylamide gel electrophoresis (SDS-PAGE) was used to separate proteins from different groups and markers (Bio-Rad, 4561094, USA). The proteins were then transferred to PVDF membranes (Millipore, IPVH00010, USA).

The membranes were incubated at 4 °C overnight with 1:1000 dilutions of JAK1 antibody (Santa Cruz Biotechnology, sc-376996, USA), STAT3 antibody (Abcam Co., EPR787Y, UK), JNK antibody (Santa Cruz Biotechnology, sc-7345, USA), ERK1 antibody (Santa Cruz Biotechnology, sc-271270, USA), and p38 antibody (Biorbyt, orb127559, USA), respectively, then incubated at room temperature for 1 h with 1:2000 dilutions of HRP-linked anti-rabbit antibody (Cell Signaling Technology, 7074, USA). The Immobilon Western HRP substrate (Thermo Fisher Scientific, 32106, USA) and ChemiDoc MP Imaging System (Bio-Rad, USA) were applied to record the band intensity of phosphorylated JAK1, STAT3, JNK, ERK, and p38 proteins. Image Lab software (Bio-Rad, USA) performed protein normalization against β-actin (Cell Signaling Technology, 4970, USA).

### Statistical analysis

The results were presented as the mean alongside the standard error (SE) (*n* = 6). To compare groups with data that follows a normal distribution, statistical evaluations were executed using a one-way ANOVA, followed by post hoc comparisons employing Tukey’s test. The statistical analysis was conducted using GraphPad Prism Software. Statistical significance was determined by a *p* value threshold of less than 0.05.

## Results

### ESOM and CANA ameliorated liver function in MTX-intoxicated rats

**Fig. 2:**
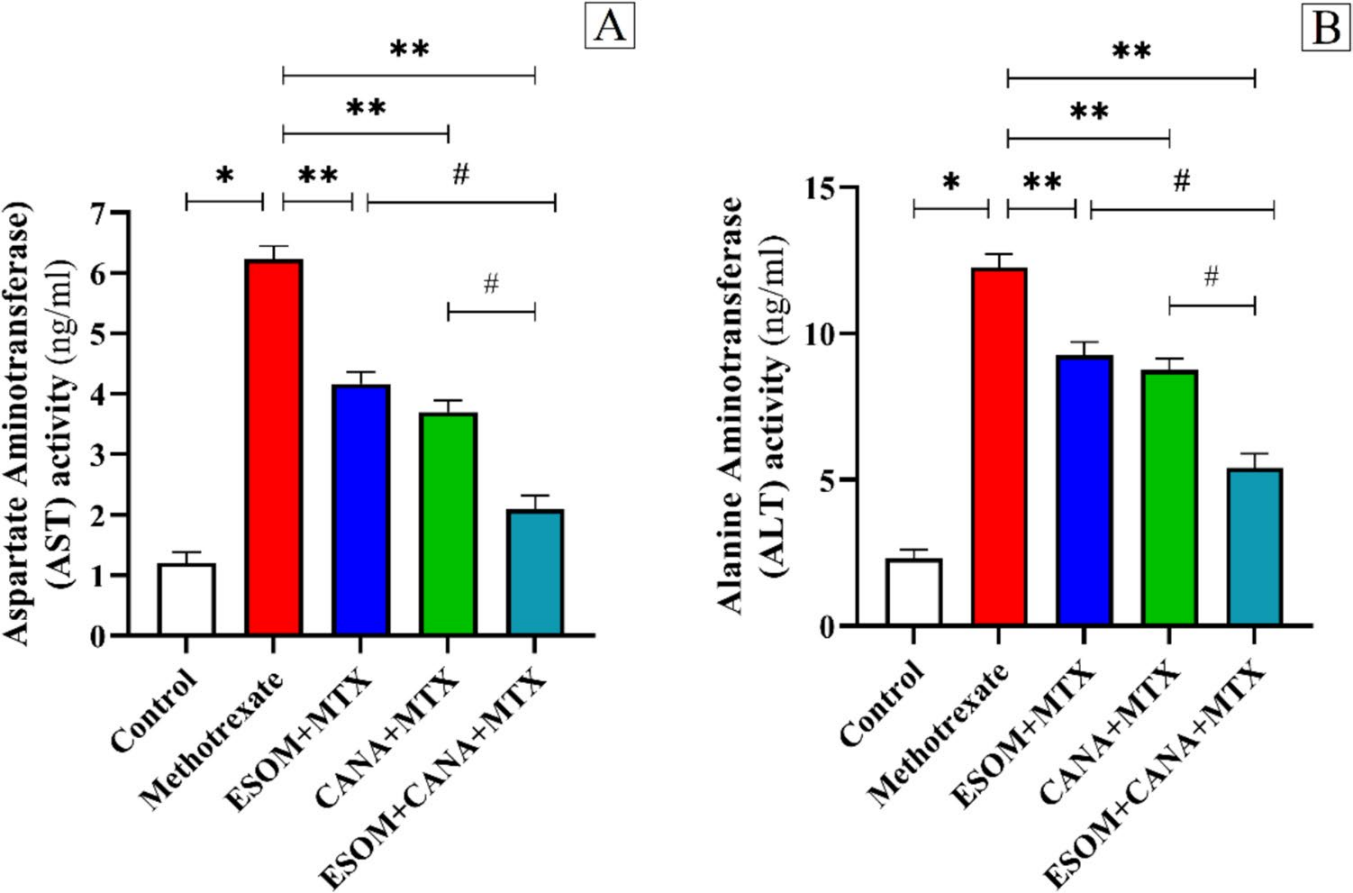
ESOM and CANA ameliorated liver function in MTX-intoxicated rats. Effect of ESOM (30 mg/kg) and CANA (30 mg/kg) on **A** AST and **B** ALT. Data are shown as mean ± SE (*n* = 6). *, **, ^#^ indicate significant variations among the control group, MTX group, and ESOM and CANA + MTX group, respectively, applying one-way ANOVA test at a *p* value < 0.01 followed by Tukey

To assess the influence of MTX on hepatic function, serum concentrations of AST and ALT were quantified. Administration of MTX (20 mg/kg, i.p.) significantly compromised liver function, as demonstrated by substantial elevations in AST and ALT levels, which increased by 4.18-fold and 4.28-fold, respectively, compared to the control group’s baseline levels (*p* < 0.01). On the contrary, notable amelioration in liver function was observed in the group pretreated with ESOM (30 mg/kg), where AST and ALT levels decreased by 0.33-fold and 0.24-fold, respectively, relative to the MTX group (*p* < 0.01). Additionally, pretreatment with CANA at 30 mg/kg resulted in reductions in AST and ALT levels by 0.40-fold and 0.28-fold, respectively, with respect to the MTX group (*p* < 0.01). Besides, the group pretreated with a combination of ESOM and CANA exhibited even more significant reductions in AST and ALT levels by 0.66-fold (*p* < 0.05) and 0.55-fold (*p* < 0.01), respectively, compared to the MTX group. Furthermore, the combination of ESOM and CANA also led to additional decreases in AST and ALT levels, by 0.49-fold and 0.41-fold, respectively, compared to the ESOM pretreated group, and by 0.43-fold and 0.38-fold, respectively, compared to the CANA pretreated group (*p* < 0.01), as illustrated in Figure 2A, B.

**Fig. 3:**
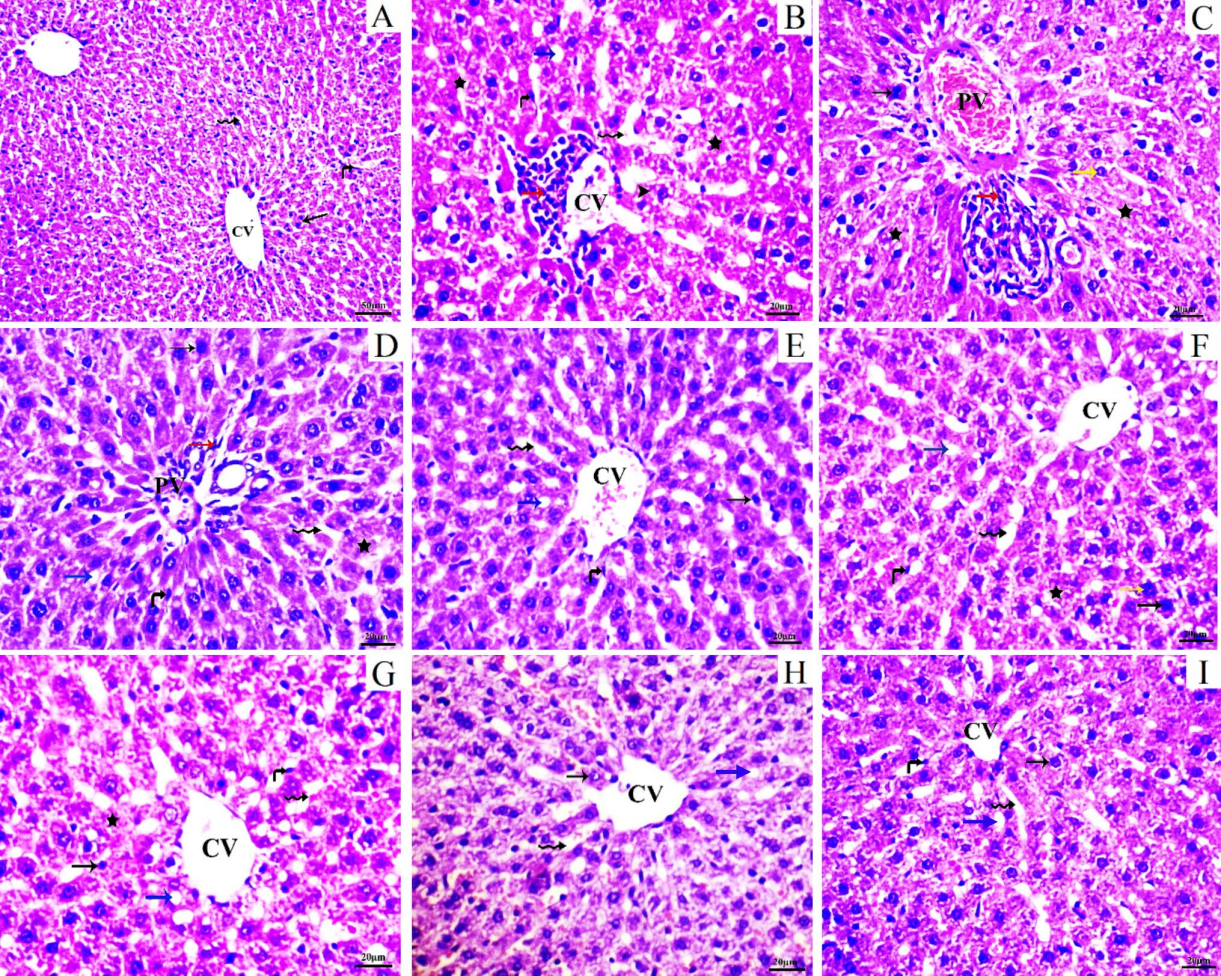
Liver sections from different experimental groups. The control group (**A**) exhibits the typical histologic structure of the central vein (CV) and surrounding hepatocytes (thin arrow) with sinusoids (wavy arrows) and Kupffer cells (curved arrow). The MTX group (**B**) shows nuclear degradation (arrowhead), disruption of hepatic cells, necrosis (star), marked fatty degeneration (blue arrow), and dilated sinusoids (wavy arrow), with the CV showing a compromised endothelial layer and lymphatic cell infiltration (red arrow). **C** Karyolysis (yellow arrow), chromatin condensation (pyknosis) (thin arrow), portal vein (PV) congestion, and polymorphonuclear leukocytes (red arrow). The ESOM pretreated group (**D**, **E**) shows inflammatory cells (red arrow) around the portal vein (PV), moderate hepatocyte degeneration (star), and fatty degeneration (blue arrow), while the CV, hepatic sinusoid (wavy arrow), and Kupffer cells (curved arrow) retain a standard pattern. The CANA pretreated group (**F**, **G**) reveals mild cell degradation (star) and fatty degeneration (blue arrow), with the CV, hepatic sinusoids (wavy arrow), and Kupffer cells (curved arrow) maintaining better morphology. The combination of ESOM and CANA pretreated group (**H**, **I**) indicates minimal hepatocyte degeneration (blue arrow), with the hepatic cords (thin arrow), sinusoids (wavy arrow), and Kupffer cells (curved arrow) exhibiting a typical arrangement

Histological analysis was performed on liver sections from various experimental groups. Liver sections from the control group exhibited no histological alterations and displayed a typical central vein, hepatocyte arrangement, sinusoids, and Kupffer cells (Figure 3A). Conversely, liver sections from the MTX group showed marked alterations in lobular structure and nuclear degradation in certain areas. There was a disruption hepatic cells arrangement, along with necrosis, fatty degeneration, karyolysis, and chromatin condensation (pyknosis). Additionally, there was expansion and congestion of the hepatic portal vein (PV). Polymorphonuclear leukocytes were observed infiltrating the portal region, and the central vein (CV) exhibited a compromised endothelial layer and infiltration of lymphatic cells, as well as dilated sinusoids (Fig. 3B, C). Liver sections from rats pretreated with ESOM at a dosage of 30 mg/kg displayed inflammatory cells surrounding the portal vein (PV), coupled with moderate hepatocytes degeneration and fatty degeneration. The central vein, hepatocytes, portal components, hepatic sinusoids, and Kupffer cells maintained their typical structure and appearance (Fig. 3D, E). Additionally, rats pretreated with CANA at a dosage of 30 mg/kg exhibited mild cellular degradation, fatty degeneration, and lymphatic infiltration. The hepatic vein was less congested, and the morphology of hepatocytes and sinusoids was well-preserved (Fig. 3F, G). Furthermore, rats pretreated with a combination of ESOM and CANA showed few inflammatory cell infiltrations around the portal triads and central veins, accompanied by minimal hepatocyte degeneration (Fig. 3H, I). The severity of histopathological changes in various experimental groups is detailed in Table 2.

**Table 2:** The severity of histopathological changes of various experimental groups

Histopathological alterations	Congestion	Lymphatic infiltration	Degeneration (pyknosis, karyolysis, necrosis)	Fatty degeneration
Groups				
Control	–	–	–	–
MTX group	+ + +	+ + +	+ + +	+ + +
ESOM (30 mg/kg) + MTX	+ +	+ +	+ +	+ +
CANA (30 mg/kg) + MTX	+ +	+ +	+ +	+ +
ESOM + CANA + MTX	+	+	+	+

(--) indicates nil (0–25%), (+) indicates mild (25–50%), (++) indicates moderate (50–75%), and (+++) indicates severe (75–100%)

### ESOM and CANA modulated AMPK activity

**Fig. 4:**
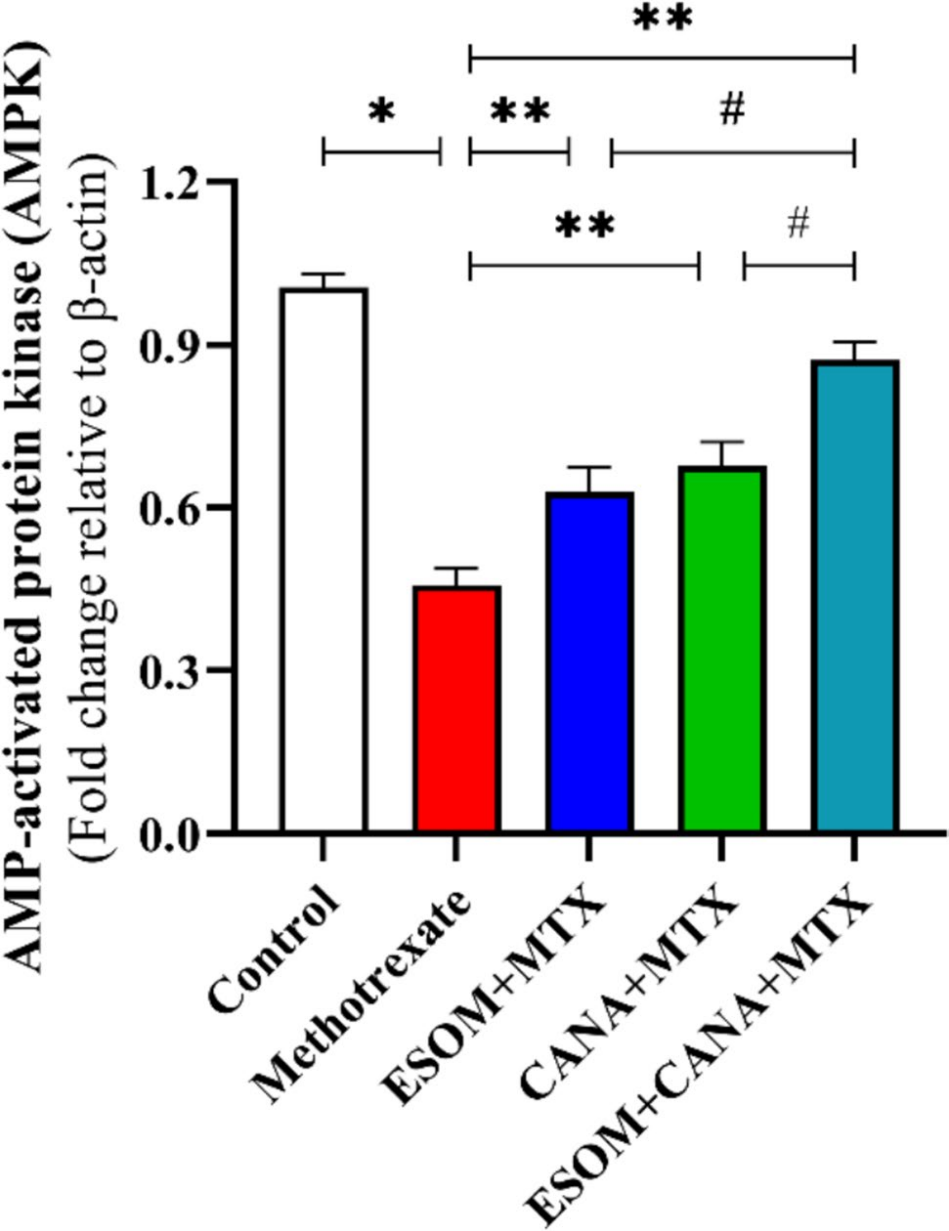
Modulation of AMPK activity by ESOM and CANA in MTX-intoxicated rats. The effects of ESOM and CANA on various groups were analyzed by measuring the relative expression of the AMPK gene using qRT-PCR. Data are presented as mean ± SE (*n* = 6). *, **, and ^#^ denote significant variations among the control group, MTX group, and the group treated with both ESOM and CANA + MTX, respectively. A one-way ANOVA test was conducted, yielding a *p* value of less than 0.01, and subsequent comparisons were made using Tukey’s post hoc test

The administration of MTX caused a notable decrease in AMPK gene expression, reducing it by 0.55-fold compared to the control group (*p* < 0.01). In contrast, pretreating with 30 mg/kg ESOM led to a 0.37-fold rise in AMPK gene expression corresponding to the MTX group (*p* < 0.05). The group that received CANA pretreatment exhibited a significant 0.48-fold elevation in AMPK gene expression corresponding to the MTX group (*p* < 0.01). Furthermore, the combined pretreatment with ESOM and CANA resulted in a substantial 0.93-fold enhancement in AMPK gene expression relative to the MTX group (*p* < 0.01). This combination also elevated AMPK gene expression by 0.40-fold compared to the ESOM pretreated group and by 0.29-fold in relation to the CANA pretreated group (*p* < 0.01) (see Fig. 4).

### Inflammatory response regulation by ESOM and CANA in MTX-intoxicated rats involved the p38MAPK/NF-κB and JAK1/STAT3 signaling pathways

To evaluate whether ESOM and CANA could mitigate the inflammatory response in hepatic cells triggered by MTX, western blotting and immunohistochemical staining techniques were utilized. These techniques measured the expression levels of proteins such as hepatic p-p38MAPK, p-JNK, p-ERK1, and NF-κB p65.

**Fig. 5:**
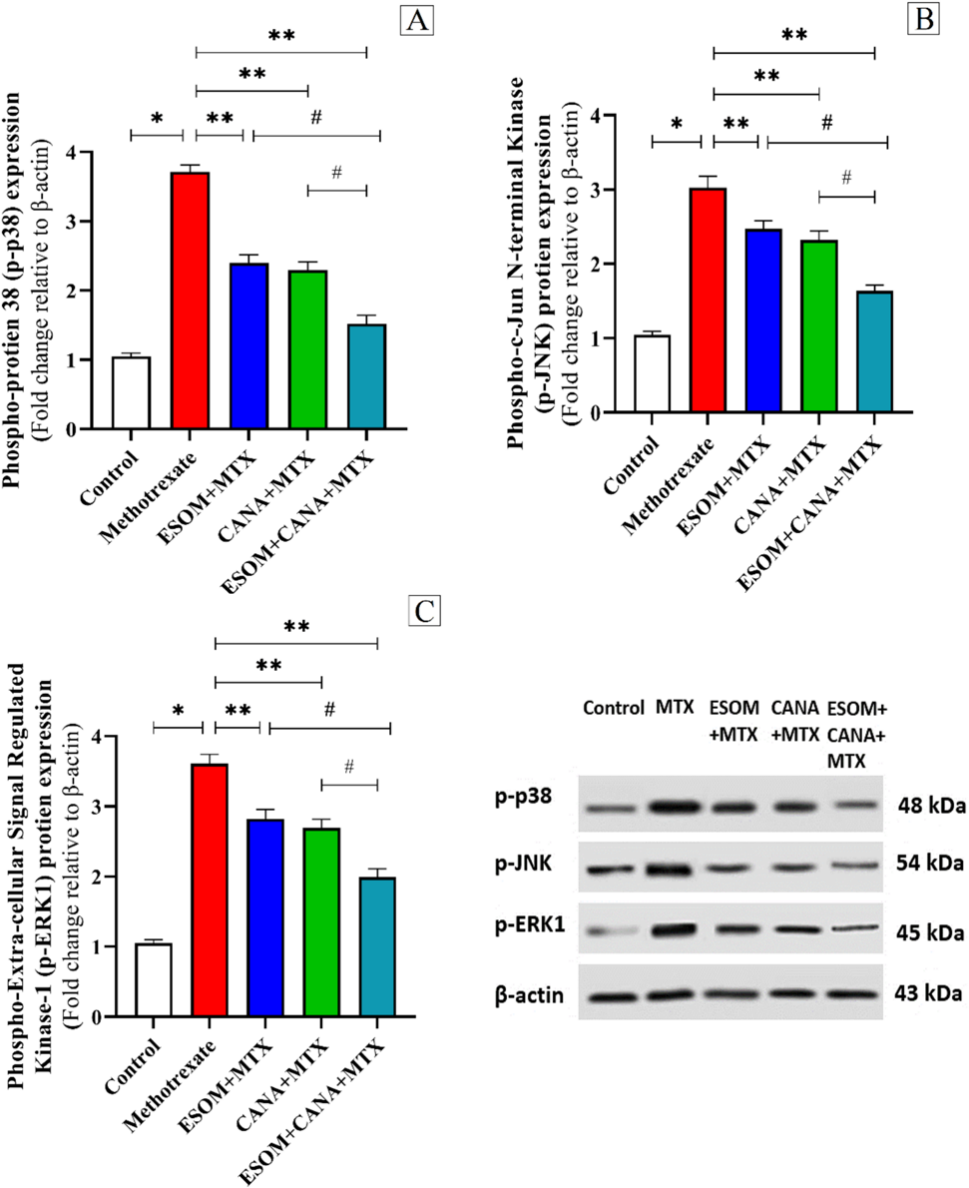
Regulation of inflammatory response by ESOM and CANA through the phosphorylated p38MAPK/JNK/ERK1 signaling pathway in MTX-intoxicated rats. The anti-inflammatory properties of ESOM and CANA were analyzed across different groups by measuring the protein expression levels of **A** p-p38, **B** p-JNK, and **C** p-ERK1 using western blotting. Data are shown as mean ± SE (*n* = 6). *, **, ^#^ indicate significant variations among the control group, MTX group and ESOM and CANA + MTX group, respectively, applying one-way ANOVA test at a *p* value < 0.01 followed by Tukey

**Fig. 6:**
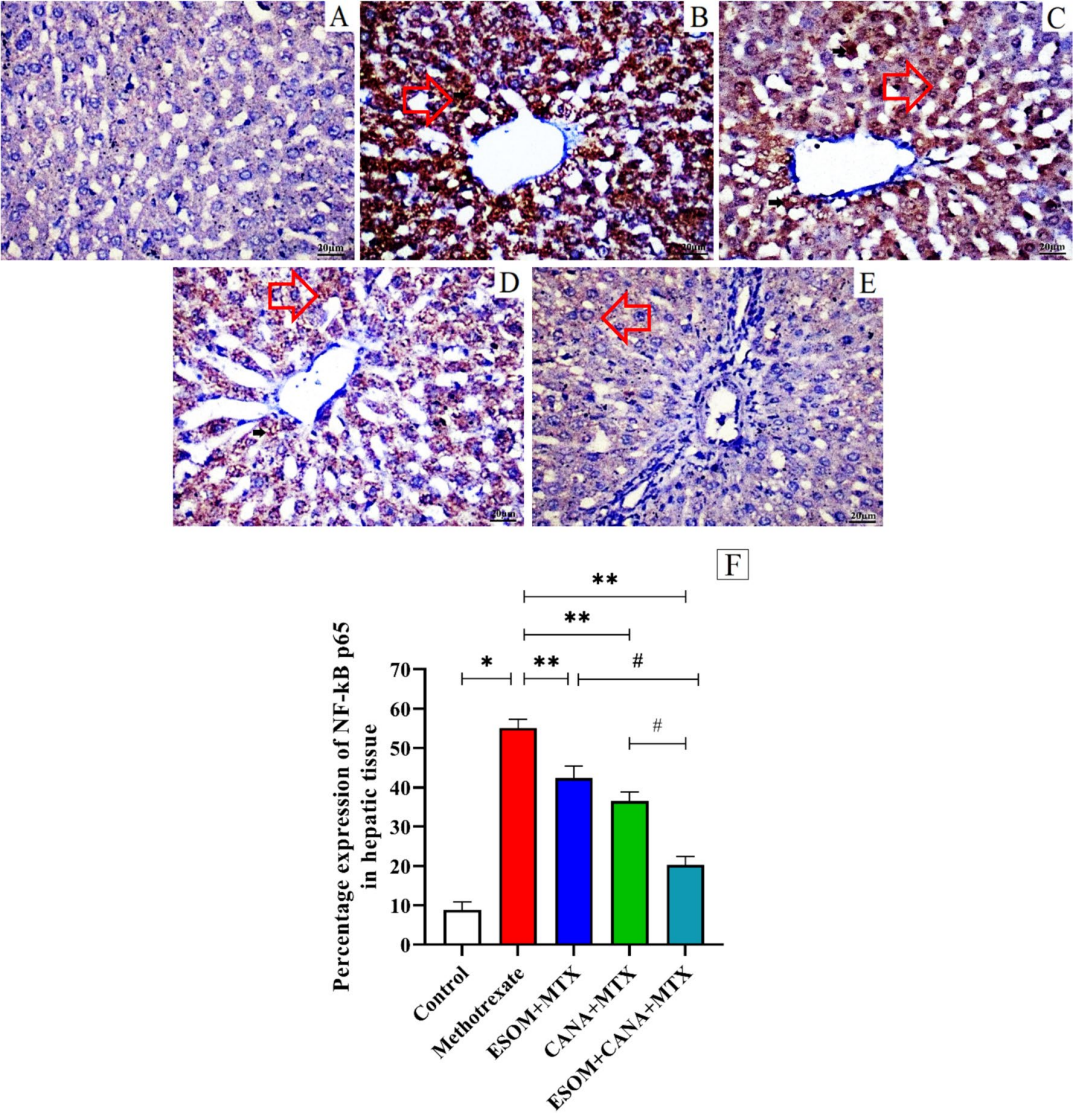
ESOM and CANA decreased hepatic NF-kB p65 expression level in MTX-injected rats. Microscopic images of liver sections from **A** control group demonstrating none to few immunoexpression of NF-kB p65; **B** rats treated with MTX exhibit marked and diffuse immunoexpression in cytoplasm of hepatocytes (arrowhead); **C** MTX-intoxicated rats pretreated with ESOM (30 mg/kg) demonstrating moderate perivascular immunopositive staining in the cytoplasm of hepatocytes (arrowhead); **D** MTX-injected rats pretreated with CANA (30 mg/kg) showing mild immunopositive expression in the cytoplasm of hepatocytes (arrowhead); and **E** MTX-injected rats pretreated with a combination of ESOM and CANA showing minimal immunopositive expression in the cytoplasm to occasional positive cells (arrowhead) (scale bar, 20). **F** The influence of ESOM and CANA on hepatic NF-kB p65 expression level. Data are expressed as mean ± SEM (*n* = 6). A significance level of *p* < 0.01. The * indicates significance in relation to the normal control group, ** shows significance relative to the MTX group, and ^#^ shows a significant difference in comparison to ESOM and CANA pretreated group

MTX treatment resulted in substantial increases in the expression levels of the proteins p-p38MAPK, p-JNK, p-ERK1, and NF-κB p65, by 2.56-, 1.9-, 2.46-, and 5.23-fold, respectively, compared to the control group (*p* < 0.01). In the group pretreated with ESOM (30 mg/kg), there were notable declines in the expression levels of the proteins p-p38MAPK, p-JNK, p-ERK1, and NF-κB p65 by 0.35-fold (*p* < 0.01), 0.18-fold (*p* < 0.05), 0.21-fold (*p* < 0.05), and 0.23-fold (*p* < 0.01), respectively, in relation to the MTX group (*p* < 0.01). Similarly, the CANA (30 mg/kg) pretreated group revealed considerable decreases in the protein levels of hepatic p-p38MAPK, p-ERK1, p-JNK, and NF-κB p65 by 0.38-fold (*p* < 0.01), 0.23-fold (*p* < 0.05), 0.25-fold (*p* < 0.05), and 0.33-fold (*p* < 0.01), respectively, compared to the MTX group (*p* < 0.01). Besides, the group pretreated with both ESOM and CANA exhibited even greater decreases in the protein levels of hepatic p-p38MAPK, p-ERK1, p-JNK, and NF-κB p65 by 0.59-, 0.46-, 0.44-, and 0.63-fold, respectively, compared to the MTX group (*p* < 0.01). Additionally, the combination of ESOM and CANA decreased the protein levels of hepatic p-p38MAPK, p-ERK1, p-JNK, and NF-κB p65 by 0.36-, 0.34-, 0.29-, and 0.52-fold (*p* < 0.05), respectively, compared to the ESOM pretreated group, and by 0.34-, 0.29-, 0.26-, and 0.44-fold (*p* < 0.05), respectively, compared to the CANA pretreated group, as shown in Fig. 5A–C and Fig. 6A–F.

**Fig. 7:**
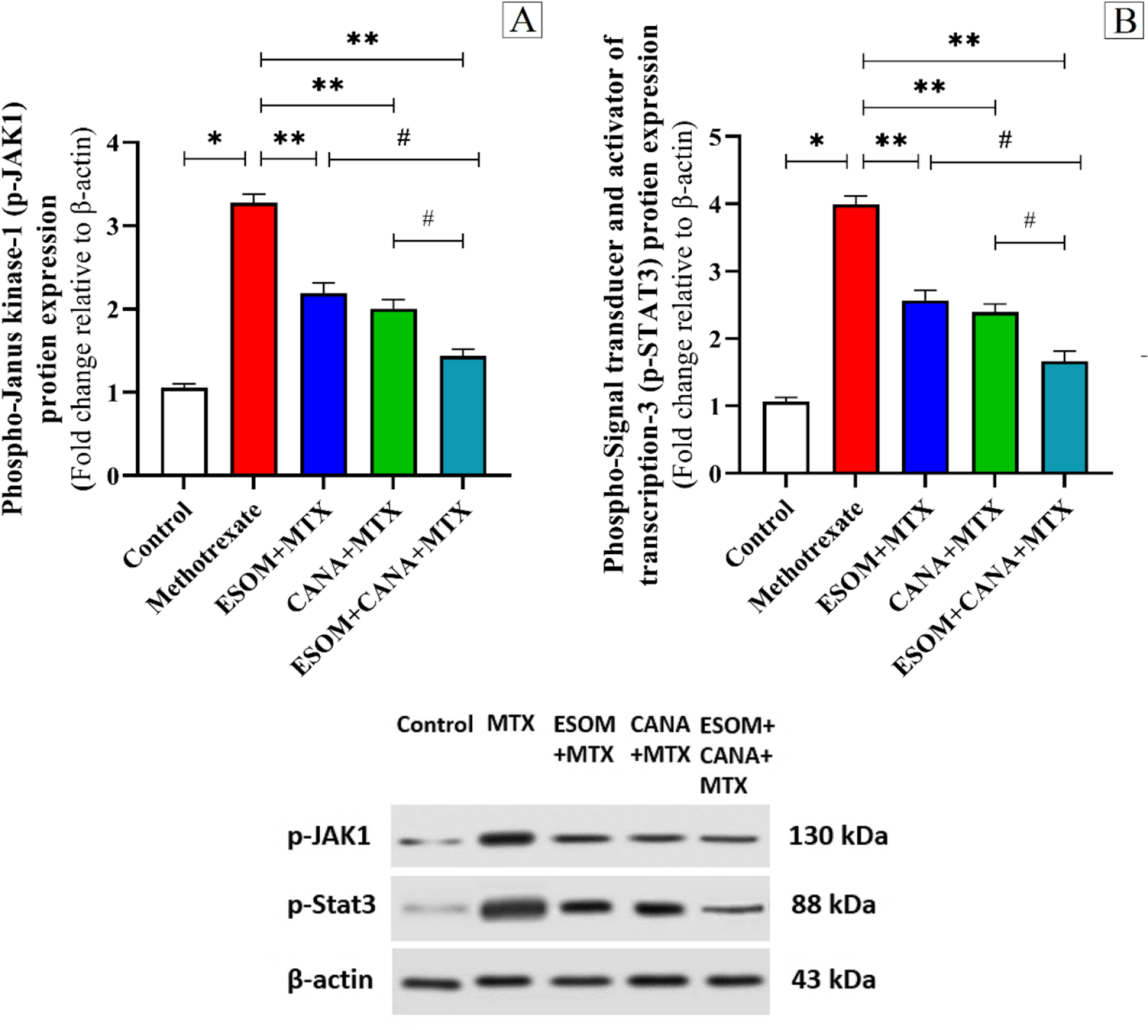
The regulation of the p-JAK1/p-STAT3 signaling pathway by ESOM and CANA. To verify the effect of ESOM and CANA on various groups, we measured **A** the relative protein expression of p-JAK1 via western blot analysis and **B** the relative protein expression of p-STAT3 using western blot analysis. Data are shown as mean ± SE (*n* = 6). *, **, ^#^ indicate a significant difference between the control group, MTX group, and ESOM and CANA + MTX group, respectively, using one-way ANOVA test at a *p* value < 0.01 followed by Tukey

The administration of MTX significantly elevated the levels of p-JAK1 and p-STAT3 proteins, increasing them by factors of 2.11 and 2.76, respectively, in relation to the control group (*p* < 0.01). Inversely, pretreatment with 30 mg/kg ESOM caused a decline in p-JAK1 and p-STAT3 protein expression by factors of 0.33 and 0.35, respectively, compared to the MTX group (*p* < 0.01). In the group pre-treated with CANA, there were notable reductions in p-JAK1 and p-STAT3 protein levels by factors of 0.38 and 0.40, respectively, corresponding to the MTX group (*p* < 0.01). Furthermore, the combined pretreatment with ESOM and CANA resulted in even more substantial decreases in p-JAK1 and p-STAT3 protein expression by factors of 0.56 and 0.58, respectively, compared to the MTX group (*p* < 0.01). This combination pretreatment also reduced p-JAK1 and p-STAT3 protein levels by factors of 0.34 and 0.35, respectively, corresponding to the ESOM pretreatment group, and by factors of 0.28 and 0.30, respectively, corresponding to the CANA pretreatment group (*p* < 0.05) (see Fig. 7A, B).

**Fig. 8:**
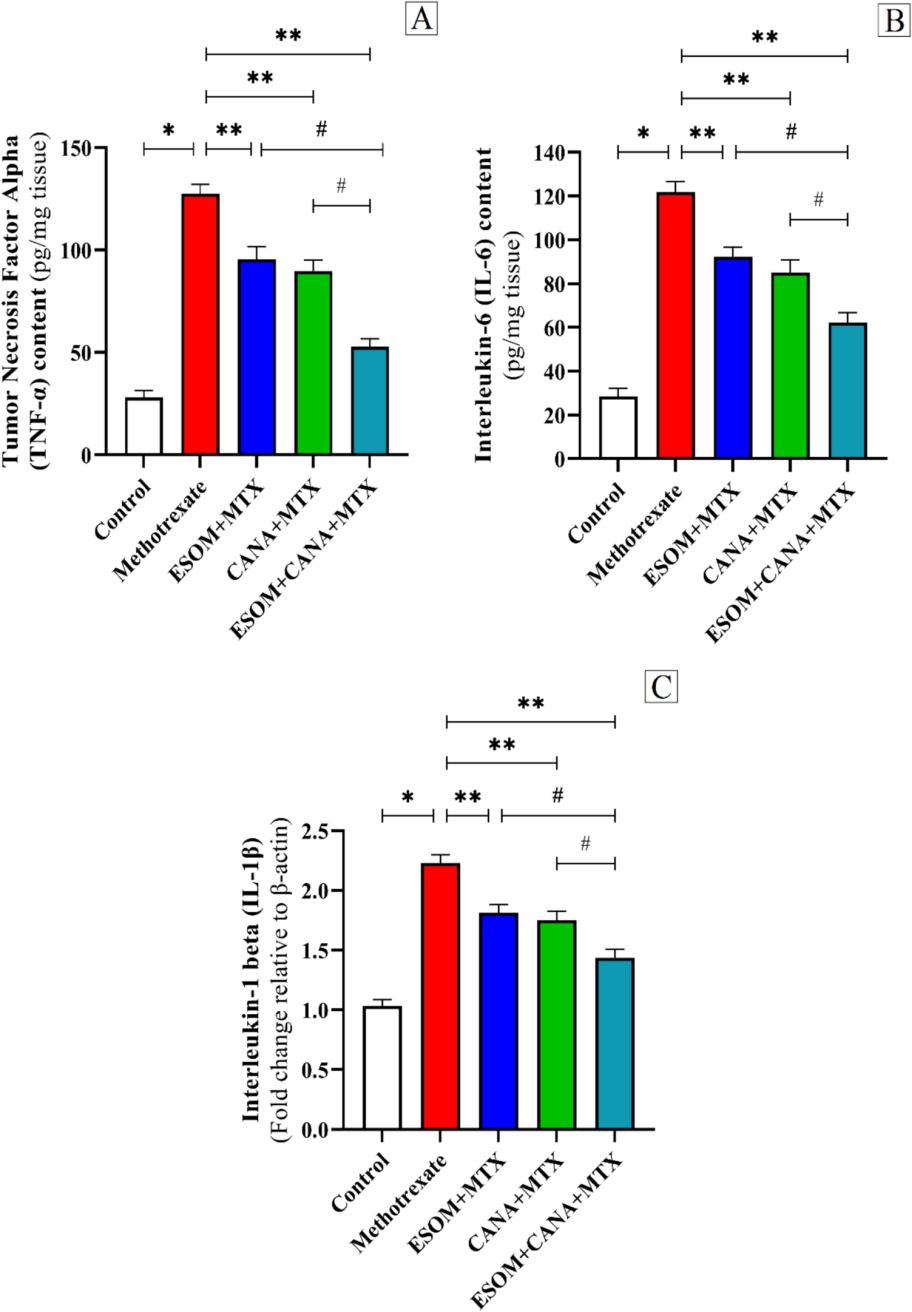
ESOM and CANA reduced cytokine levels in rats treated with MTX. To measure the effects of ESOM and CANA in different groups, we measured **A** TNF-α levels through ELISA, **B** IL-6 levels via ELISA, and **C** IL-1β expression levels using qRT-PCR. Data are shown as mean ± SE (*n* = 6). *, **, ^#^ indicate significant variations among the control group, MTX group, and ESOM and CANA + MTX group, respectively, applying one-way ANOVA test at a *p* value < 0.01 followed by Tukey

The study demonstrated that MTX administration significantly increased the mRNA expression levels of IL-1β, IL-6, and TNF-α by factors of 1.15, 3.27, and 3.56, respectively, compared to the control group (*p* < 0.01). Conversely, pretreatment with 30 mg/kg ESOM resulted in reductions of IL-1β, IL-6, and TNF-α mRNA expression by factors of 0.18, 0.24, and 0.25, respectively, in relation to the MTX group (*p* < 0.01). Similarly, pretreatment with 30 mg/kg CANA led to notable decreases in IL-1β, IL-6, and TNF-α mRNA expression by factors of 0.21, 0.30, and 0.29, respectively, with respect to the MTX group (*p* < 0.01). Furthermore, the combined pretreatment with ESOM and CANA resulted in even greater reductions in IL-1β, IL-6, and TNF-α mRNA expression by factors of 0.35, 0.48, and 0.58, respectively, corresponding to the MTX group (*p* < 0.01). Additionally, this combination treatment decreased IL-1β, IL-6, and TNF-α mRNA expression by factors of 0.20, 0.32, and 0.44, respectively, compared to the ESOM pretreated group (*p* < 0.01), and by factors of 0.17 (*p* < 0.05), 0.26 (*p* < 0.05), and 0.41 (*p* < 0.01), respectively, with regard to the CANA pretreated group (refer to Fig. 8A–C). These data suggest that the anti-inflammatory properties of ESOM and CANA may play a crucial role in their protective effects against MTX-induced hepatotoxicity.

### ESOM and CANA mitigated MTX-induced oxidative stress by influencing the Nrf2/HO-1 pathway

To evaluate the antioxidant capabilities of ESOM and CANA, levels of MDA, MPO, iNOS, GSH, Nrf2, and HO-1 were measured in liver homogenates.

**Fig. 9:**
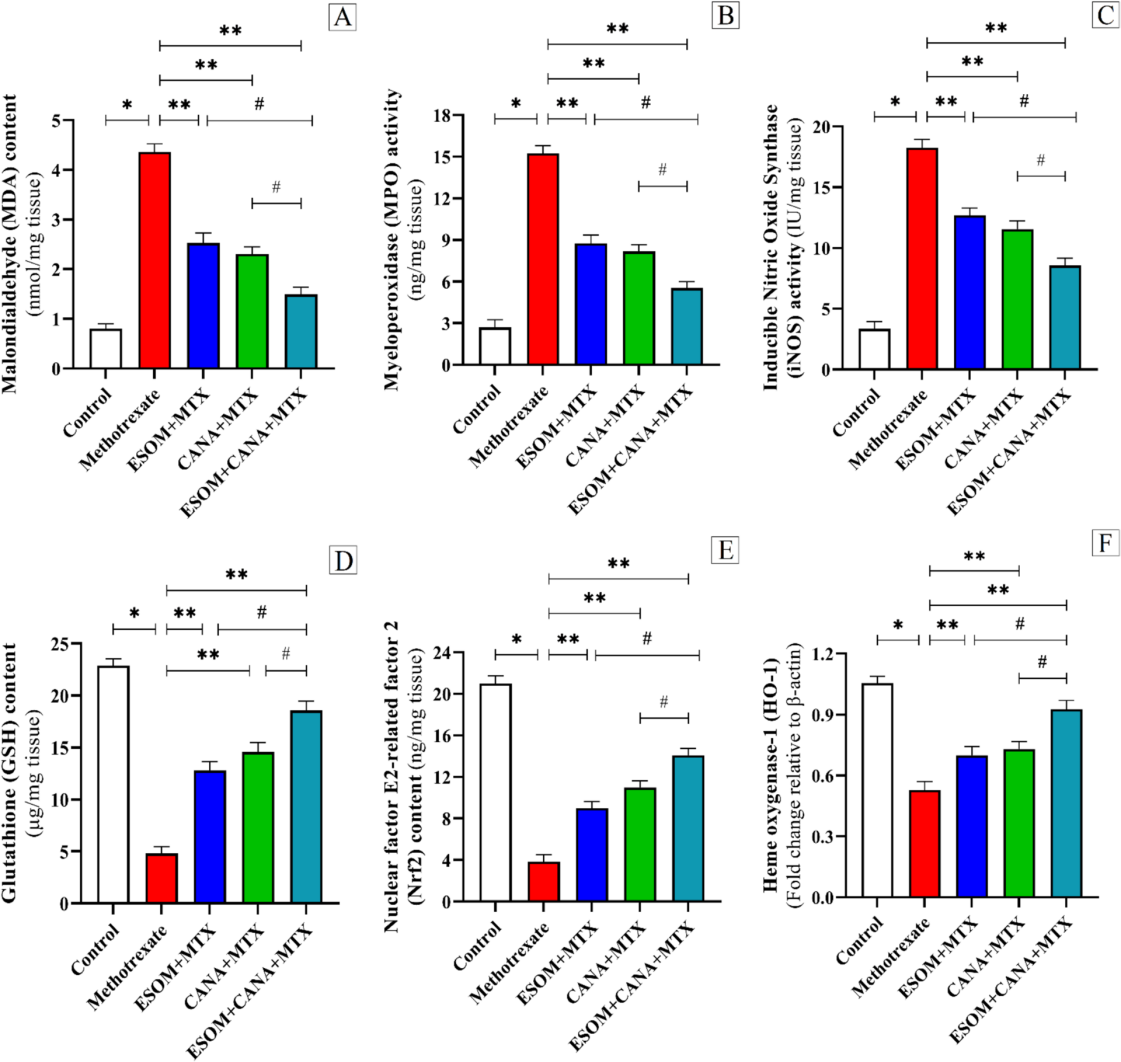
ESOM and CANA reduced MTX-induced oxidative stress through the regulation of the Nrf2/HO-1 signaling pathway. The antioxidative effects of ESOM and CANA were assessed in different groups by measuring **A** MDA levels, **B** MPO levels, **C** iNOS levels, **D** GSH levels, and **E** Nrf-2 levels using ELISA, while **F** HO-1 levels were determined using qRT-PCR. Data are shown as mean ± SE (*n* = 6). *, **, ^#^ indicate significant variations among the control group, MTX group, and ESOM and CANA + MTX group, respectively, applying one-way ANOVA test at a *p* value < 0.01 followed by Tukey

The group treated with MTX showed significant elevations in MDA, MPO, and iNOS levels, alongside notable reductions in GSH, Nrf2, and HO-1 mRNA expression, with changes of 4.45, 4.68, 4.48, 0.79, 0.81, and 0.50 times, respectively, in relation to the control group (*p* < 0.01). Conversely, the ESOM pretreated group revealed decreased MDA, MPO, and iNOS levels, with increased GSH, Nrf2, and HO-1 mRNA expression, by 0.41, 0.42, 0.30, 1.67, 1.33, and 0.32 times, respectively, compared to the MTX group (*p* < 0.01). The CANA pretreated group showed reduced MDA, MPO, and iNOS levels, with rises in GSH, Nrf2, and HO-1 mRNA expression, by 0.47, 0.46, 0.36, 2.04, 1.85, and 0.40 times, respectively, compared to the MTX group (*p* < 0.01). Additionally, the combination of ESOM and CANA pretreatment led to even more pronounced reductions in MDA, MPO, and iNOS levels, as well as greater increases in GSH, Nrf2, and HO-1 mRNA expression, by 0.65, 0.63, 0.53, 2.88, 2.66, and 0.76 times, respectively, with respect to the MTX group (*p* < 0.01). Besides, the combination pretreatment with ESOM and CANA resulted in decreases in MDA, MPO, and iNOS levels, along with increases in GSH, Nrf2, and HO-1 mRNA expression, by 0.41, 0.36, 0.32, 0.45, 0.56, and 0.33 times, respectively, compared to the ESOM pretreated group (*p* < 0.01), and by 0.35 (*p* < 0.05), 0.32 (*p* < 0.05), 0.25 (*p* < 0.05), 0.27 (*p* < 0.01), 0.28 (*p* < 0.05), and 0.26 times (*p* < 0.05), respectively, relative to the CANA pretreated group (see Fig. 9A–F).

### ESOM and CANA regulated the PI3K/Akt signaling pathway

**Fig. 10:**
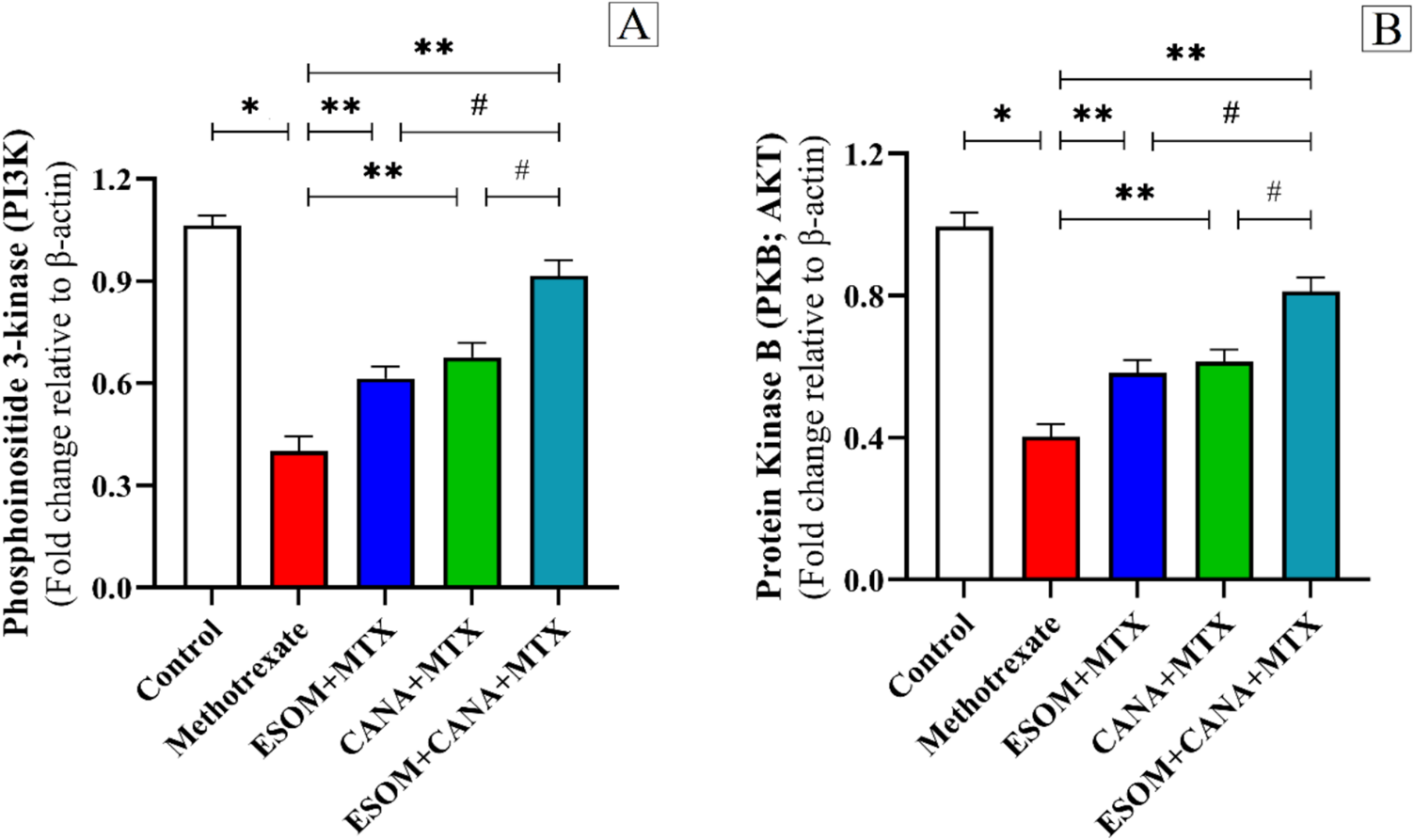
ESOM and CANA modulated the PI3K/Akt signaling pathway. To assess the effects of ESOM and CANA across different groups, we measured **A** PI3K relative gene expression via qRT-PCR, and **B** Akt relative gene expression of via qRT-PCR. Data are shown as mean ± SE (*n* = 6). *, **, ^#^ indicate significant variations among the control group, MTX group, and ESOM and CANA + MTX group, respectively, applying one-way ANOVA test at a *p* value < 0.01 followed by Tukey

Administering MTX led to a marked decrease in PI3K and Akt gene expression, reducing their levels by 0.62 and 0.59 times, respectively, with respect to the control group. Conversely, pretreatment with ESOM elevated PI3K and Akt gene expression by 0.52 and 0.45 times, respectively, compared to the MTX group (*p* < 0.05). The group pretreated with CANA showed significant increases in PI3K and Akt gene expression, rising by 0.67 and 0.52 times, respectively, compared to the MTX group (*p* < 0.01). Moreover, combined pretreatment with ESOM and CANA resulted in substantial increases in PI3K and Akt gene expression, by 1.27 and 1.02 times, respectively, relative to the MTX group (*p* < 0.01). Additionally, this combination treatment enhanced PI3K and Akt gene expression by 0.49 and 0.39 times, respectively, corresponding to the ESOM pretreatment group, and by 0.35 and 0.32 times, respectively, compared to the CANA pretreatment group (*p* < 0.01) (refer to Fig. 10A, B).

### ESOM and CANA reduced MTX-induced hepatic tissue apoptosis

**Fig. 11:**
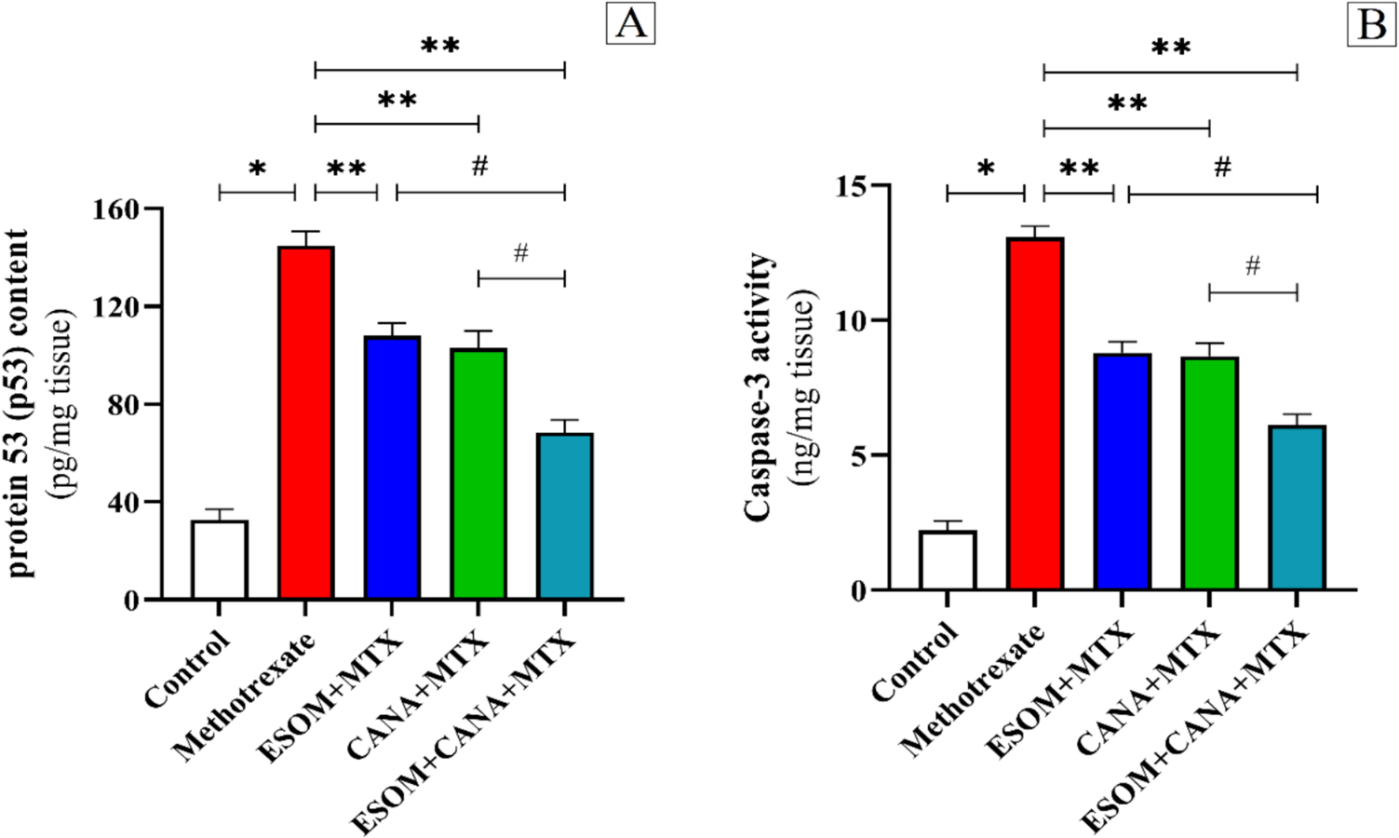
ESOM and CANA pretreatment mitigated apoptosis in hepatic tissue induced by MTX. The antiapoptotic effects of ESOM and CANA were assessed in different groups by measuring **A** p53 levels using ELISA and **B** caspase-3 levels determined by ELISA. Data are shown as mean ± SE (*n* = 6). *, **, ^#^ indicate significant variations among the control group, MTX group, and ESOM and CANA + MTX group, respectively, applying one-way ANOVA test at a *p* value < 0.01 followed by Tukey

**Fig. 12:**
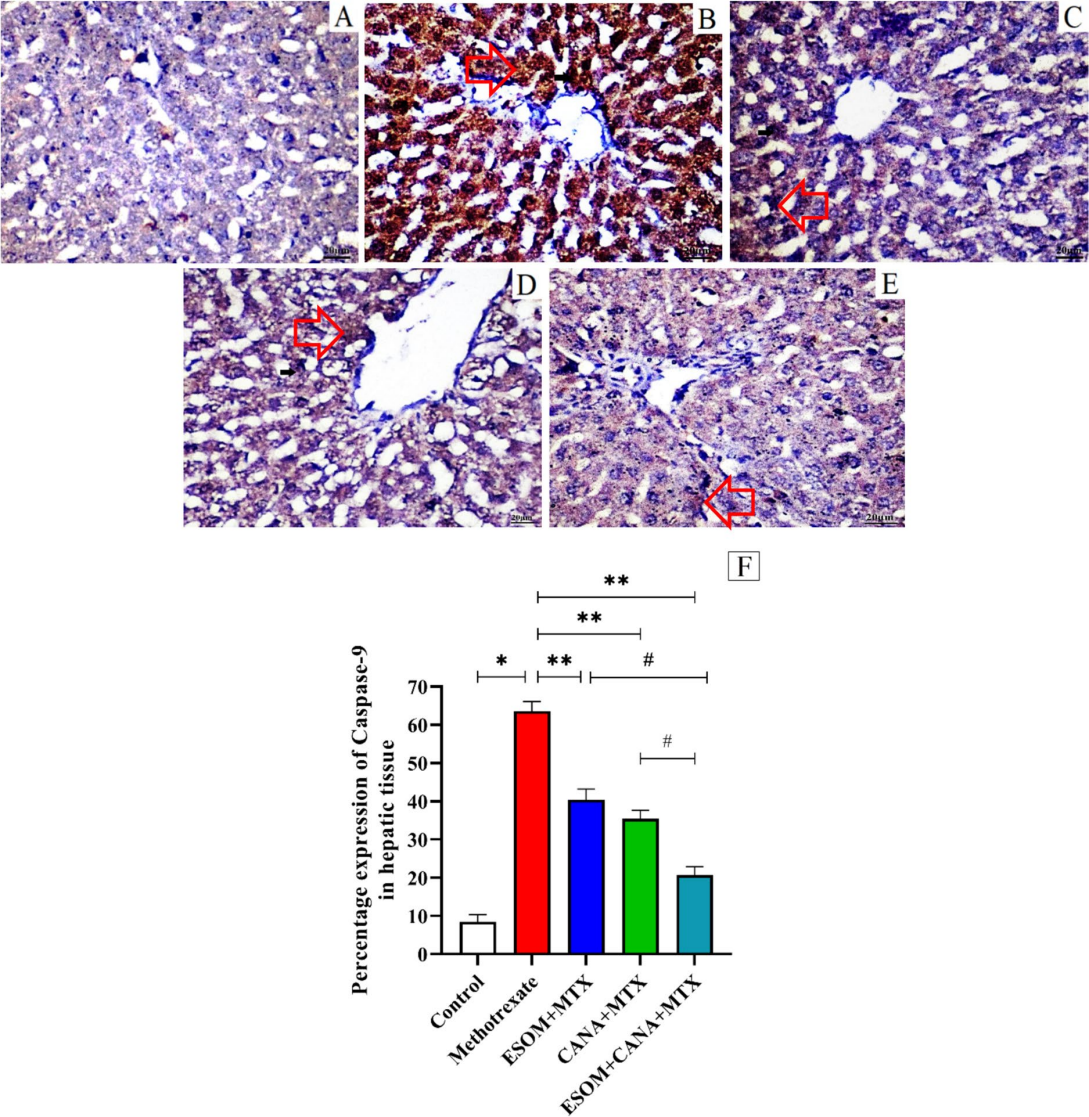
ESOM and CANA decreased hepatic caspase-9 expression level in MTX-injected rats. Microscopic images of liver sections from **A** control group demonstrating none to few immunoexpression of caspase-9; **B** rats treated with MTX exhibit pronounced and widespread immunoexpression in cytoplasm of hepatocytes (arrowhead); **C** MTX-intoxicated rats pretreated with ESOM (30 mg/kg) demonstrating moderate perivascular immunopositive staining in the cytoplasm of hepatocytes (arrowhead); **D** MTX-injected rats pretreated with CANA (30 mg/kg) showing mild immunopositive expression in the cytoplasm of hepatocytes (arrowhead); and **E** MTX-injected rats pretreated with a combination of ESOM and CANA showing minimal immunopositive expression in the cytoplasm to occasional positive cells (arrowhead) (scale bar, 20). **F** The influence of ESOM and CANA on hepatic caspase-9 expression level. Results are expressed as mean ± SEM (*n* = 6). A significance level of *p* < 0.01. The * reveals significance in relation to the normal control group, ** shows significance relative to the MTX group, and ^#^ shows a significant difference in comparison to ESOM and CANA pretreated group

Our findings confirm that MTX treatment triggers apoptosis, as indicated by the significant upregulation of p53 and caspase-3 levels, along with caspase-9 protein expression, which increased by factors of 3.42, 4.94, and 6.54, respectively, compared to the control group (*p* < 0.01). In contrast, pretreatment with ESOM reduced the extent of MTX-induced hepatic apoptosis. In the ESOM pretreated group, the levels of p53 and caspase-3, and the expression of caspase-9 protein decreased by factors of 0.25, 0.32, and 0.36, respectively, compared to the MTX group (*p* < 0.01). Similarly, the CANA pretreated group showed notable reductions in the levels of p53 and caspase-3, and the expression of caspase-9 protein by factors of 0.28, 0.33, and 0.44, respectively, compared to the MTX group (*p* < 0.01). Furthermore, the combination of ESOM and CANA pretreatment led to substantial decreases in the levels of p53 and caspase-3, and the expression of caspase-9 protein by factors of 0.52, 0.53, and 0.67, respectively, compared to the MTX group (*p* < 0.01). Additionally, the combined ESOM and CANA pretreatment resulted in reductions in the levels of p53 and caspase-3, and the expression of caspase-9 protein by factors of 0.36, 0.30, and 0.48, respectively, with regard to the ESOM pretreated group, and by factors of 0.33, 0.29, and 0.41, respectively, with respect to the CANA pretreated group (*p* < 0.01) (see Figs. 11A, B and 12A–F).

## Discussion

The antifolate metabolite MTX is well known for its effectiveness in the treatment of various cancers and autoimmune diseases. Nevertheless, its ability to induce toxicity in several organs, with a particular emphasis on hepatotoxicity characterized by elevated ROS production and the activation of inflammation and cell death pathways, complicates its use (Marin et al. 2022). This limitation emphasizes the critical need for developing effective strategies to alleviate the hepatotoxic effects associated with MTX.

In line with prior studies (Azadian et al. 2023; Pradhan et al. 2023), this research confirms the hepatotoxic effects of MTX, as demonstrated by raised serum transaminase levels (AST and ALT) and histopathological abnormalities within the liver tissue. The rise in liver enzyme levels in the bloodstream is typically indicative of hepatocellular damage, congestion in the central vein and portal vein, as well as disruption of hepatic cell arrangement. This is often accompanied by necrosis, fatty degeneration, karyolysis, and chromatin condensation, which are common effects of MTX administration. In contrast, the administration of ESOM and CANA have shown a protective effect on the liver, mitigating MTX-induced hepatotoxicity. This is reflected in the decreased levels of serum markers and the amelioration of liver tissue damage in histological examinations.

AMP-activated protein kinase (AMPK) is recognized for its contribution to mitigating oxidative stress, boosting cellular antioxidant defenses, and modulating inflammation (Huang et al. 2022). Despite AMPK’s direct interactions with various molecular targets, numerous studies have demonstrated that AMPK activation inhibits the MAPK and NF-κB p65 signaling pathways (Wu et al. 2023) and concurrently stimulates the Nrf2 and PI3K signaling pathways (Miao et al. 2023). Moreover, AMPK directly phosphorylates proteins in the JAK1/STAT3 pathway, resulting in their inhibition (Fonseca et al. 2022). Several toxicities, such as neurotoxicity, nephrotoxicity, and hepatotoxicity, have been associated with the suppression of AMPK activity (Triningsih et al. 2021; Wu et al. 2022; El-Dessouki et al. 2024a). Conversely, pharmacological activation of AMPK has demonstrated benefits in enhancing organ structure and function by diminishing oxidative stress, inflammation, and apoptosis (Li et al. 2022a). Consequently, multiple studies have underscored AMPK's significance as a critical focus for therapeutic intervention in a variety of hepatic diseases (Fang et al. 2022, Marcondes-de-Castro et al. 2023). This study’s findings reveal that MTX treatment significantly decreased AMPK levels in liver tissues, an effect that was countered by ESOM and CANA. These results emphasize the pivotal role of AMPK in protecting against drug-induced liver damage. Furthermore, this research represents the pioneering effort to investigate the impacts of ESOM and CANA on the regulation of AMPK within a liver toxicity model induced by MTX.

Nrf2 is a crucial transcription factor integral to maintaining cellular redox balance by controlling the expression of antioxidant genes like HO-1, which safeguard against ROS-induced tissue damage (Mansouri et al. 2022). Studies have indicated that MTX negatively impacts Nrf2 levels, suggesting its potential as a target for therapeutic strategies to mitigate MTX-induced hepatotoxicity (Al-khawalde et al., 2022). Several compounds have demonstrated protective effects against MTX-induced liver injury by activating the Nrf2 signaling pathway (Al-khawalde et al. 2022; Karimi et al. 2023). In this investigation, ESOM and CANA were found to increase the levels of Nrf2, HO-1, and GSH in a dose-dependent manner while concurrently reducing levels of MDA, MPO, and iNOS. These findings indicate that ESOM and CANA may mitigate MTX-induced oxidative stress through activation of the Nrf2 pathway. Further investigation revealed a synergistic interaction between Nrf2 and AMPK, with AMPK activation enhancing Nrf2 nuclear translocation and boosting HO-1 expression (Hao and Gao 2022). This interaction underscores a notable positive correlation among these biomolecules, as evidenced by our research findings.

The MAPK family, which includes serine/threonine protein kinases, is critical for the transmission of signals that govern cellular processes such as differentiation, proliferation, survival, and apoptosis, as well as the control of inflammatory mediators (Cheng et al. 2022). Within the MAPK signaling pathway are three kinase isoforms: p38, ERK, and JNK (Abdelzaher et al. 2022). Of these, the p38MAPK pathway is particularly significant for its role in activating NF-κB, which results in the phosphorylation and nuclear translocation of the p65 subunit (Xia et al. 2022). This specific subunit of NF-κB is indispensable for the transcription of genes that participate in inflammatory responses, such as tumor necrosis factor-alpha (TNF-α), interleukin 1-beta (IL-1β), and IL-6 (Li et al. 2022b). Previous studies have established a connection between MAPKs, NF-κB, and the onset of chemotherapy-induced hepatotoxicity (Mohammed et al. 2020; AlAsmari et al. 2021). Our study corroborates these findings by showing that MTX administration markedly upregulates hepatic p38MAPK expression and increases the protein levels of NF-κB, TNF-α, IL-1β, and IL-6. These results are consistent with earlier research (Younis et al. 2021) that reported elevated p38MAPK and NF-κB levels following MTX-induced liver toxicity. In contrast, pretreatment with ESOM and CANA has been shown to significantly reduce the MTX-induced elevations in p38 MAPK, NF-κB, TNF-α, IL-1β, and IL-6 levels. These findings are in line with previous research highlighting the anti-inflammatory properties of ESOM and CANA, likely due to their ability to enhance AMPK activity (Gu et al. 2022; Shoda et al. 2023).

The JAK/STAT and MAPK signaling pathways are central in regulating apoptosis and inflammation (Haftcheshmeh et al. 2022). The JAK/STAT pathway includes three main elements: cell membrane receptors, JAK proteins, and STAT proteins (Hu et al. 2023). Upon cytokine binding to their respective receptors, JAK proteins are activated and subsequently phosphorylate tyrosine residues located on the receptors. STAT proteins then attach to these sites, undergo phosphorylation, and form homo- or heterodimers through their SH2 domain. These dimers move to the nucleus, where they attach to the promoters of target genes, thereby regulating gene expression (Valle-Mendiola et al. 2023). This suggests that extracellular cytokines are essential for triggering the activation of the JAK/STAT pathway. Consequently, inflammatory cytokines like IL-6 can serve as activators, initiating the JAK1/STAT3 pathway and increasing phosphorylation of JAK1 and STAT3 proteins (Zeng et al. 2021). Our findings also show that MTX upregulates JAK1/STAT3 protein levels, which subsequently enhances the release of inflammatory cytokines, including TNF-α, IL-1β, and IL-6. The activation of the JAK1/STAT3 pathway leads to the production of ROS, resulting in DNA or protein damage and initiating the mitochondrial pathway to facilitate apoptosis (Khashab et al. 2021). This activation probably contributed to the heightened expression of inflammatory mediators like iNOS observed in our study, aligning with previous research (A-Elgadir et al., 2024) that demonstrated syringic acid’s anti-inflammatory properties by modulating the NF-κB/COX-2/iNOS and JAK/STAT pathways in hepato-testicular inflammation induced by methyl cellosolve in rats. Pretreatment with ESOM and CANA significantly decreased JAK1/STAT3 protein expression in MTX-intoxicated rats, demonstrating their anti-inflammatory properties. This finding aligns with previous studies (Hua et al. 2023) that highlighted CANA’s ability to enhance myocardial function and reduce inflammation in a rat model of cardiac arrest by inhibiting JAK/STAT signaling.

Programmed cell death, known as apoptosis, is triggered by a variety of factors and regulated through several signaling pathways (Yuan and Ofengeim 2024). Several studies have shown a connection between MTX-induced hepatotoxicity and cellular apoptosis (Alfwuaires 2022; Khalaf et al. 2022). MTX can trigger oxidative stress through the buildup of free radicals, notably hydroxyl radicals, or by directly binding to DNA strands, both of which cause DNA damage. This damage initiates the activation of key protein kinases involved in responding to DNA damage, which leads to the phosphorylation and subsequent activation of p53 (Hassanein et al. 2023). MTX has been observed to activate p53 in various scenarios, including liver models both in vitro and in vivo. The tumor suppressor protein p53, a crucial regulator of cell death induced by MTX, governs the expression of pro-apoptotic genes. This regulation facilitates the permeabilization of the mitochondrial membrane and enhances the expression of genes that initiate apoptosis (Schmidt et al. 2022a; Wasfey et al. 2023). Extensive research has established p53 operates as a downstream effector within the PI3K/Akt signaling pathway, influencing the expression of the anti-apoptotic protein Bcl-2 (Navaei et al. 2021). Disruption of the PI3K/Akt signaling pathway is linked to liver damage, characterized by elevated ROS production and apoptosis subsequent to MTX treatment. Activation of the PI3K/Akt pathway can reduce apoptosis in hepatic cells and mitigate acute liver injury induced by MTX, thus playing a vital role in maintaining liver function (Wang et al. 2023). Research has shown that when Akt is activated, it enhances MDM2's capacity to degrade p53 by promoting its phosphorylation (Chibaya et al. 2021). Our findings, consistent with previous studies (Arab et al. 2018; Sherif et al. 2019), showed decreased PI3K and Akt expression, along with increased levels of p53, caspase-9, and caspase-3 in MTX-intoxicated rats. However, the administration of ESOM and CANA countered MTX-induced apoptosis through activation of the PI3K/Akt pathway. These results are consistent with those of Eltahir and Nazmy (2018), who found that ESOM alleviates CCl4-induced liver fibrosis induced by CCl4. This effect is achieved by controlling oxidative stress, inflammation, and apoptosis, leading to increased levels of the anti-apoptotic protein Bcl-2 and reduced levels of the pro-apoptotic protein Bax. Additionally, CANA significantly upregulated cardiac levels of PI3K/AKT and effectively mitigated cisplatin-induced apoptosis by modulating Bax, cytochrome C, and Bcl-2 protein levels (Ali et al. 2023).

## Conclusion

**Fig. 13:**
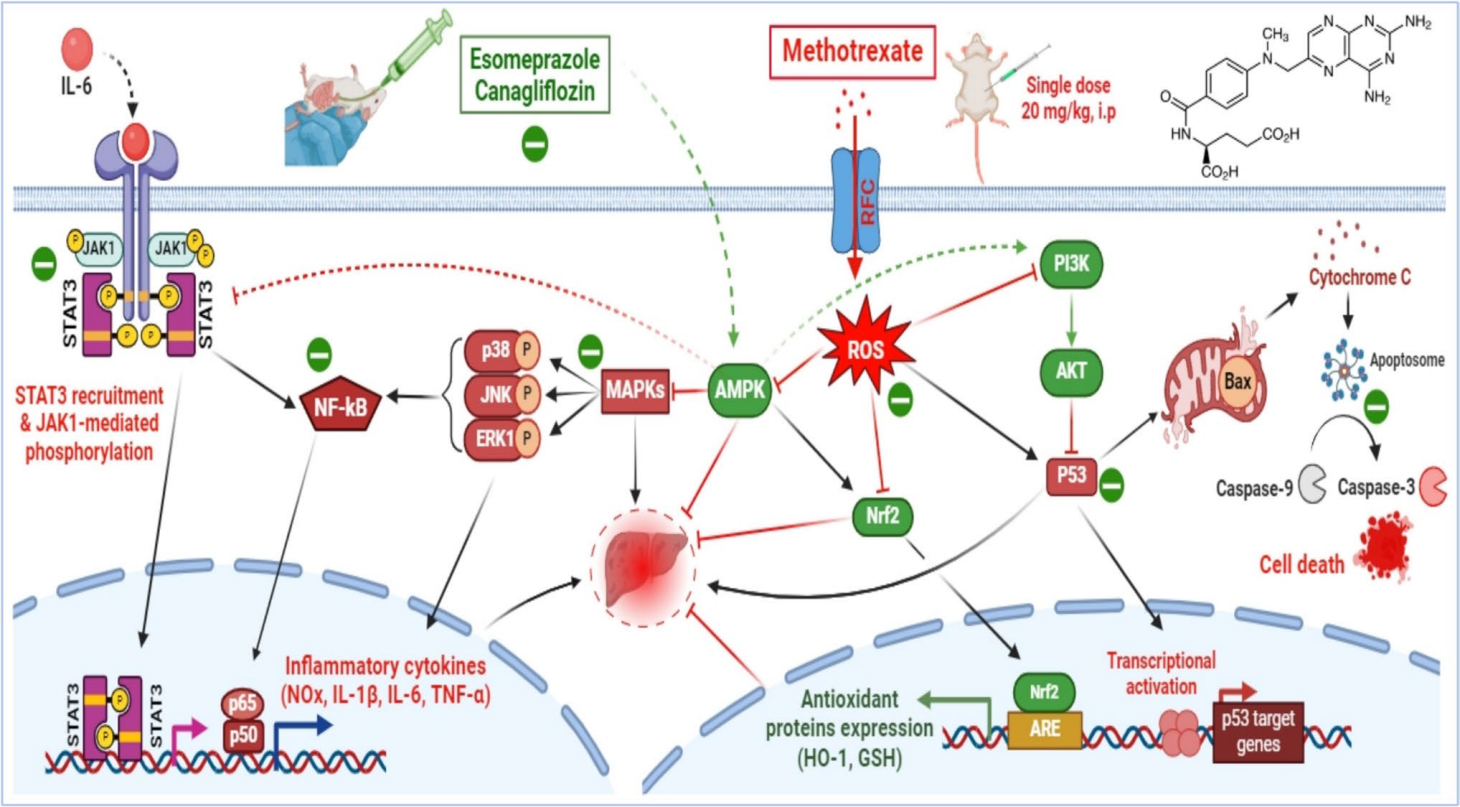
Proposed mechanism of ESOM and CANA action. In a rat model, ESOM and CANA mitigated MTX-induced hepatotoxicity by triggering AMPK activation. This activation influences the transcription of downstream genes that are crucial for managing oxidative stress, inflammation, and apoptosis

ESOM and CANA modulate AMPK activity, which is essential for maintaining cellular energy balance, reducing inflammation, and enhancing antioxidant defenses. By activating AMPK, they increase antioxidant enzymes like HO-1 and GSH and reduce oxidative stress markers such as MDA, MPO, and iNOS. These agents also suppress inflammation by inhibiting the MAPK/NF-κB p65 and JAK1/STAT3 pathways, lowering pro-inflammatory cytokines like TNF-α, IL-6, and IL-1β. Additionally, they counteract MTX-induced apoptosis via activation of the PI3K/Akt pathway, supporting cell survival and reducing apoptotic processes. The hepatoprotective effects of ESOM and CANA arise from their antioxidative, anti-inflammatory, and anti-apoptotic actions, mediated through key signaling pathways. Their combined treatment shows synergistic benefits, with significant improvements in biochemical, histopathological, and molecular outcomes (Fig. 13). While limitations include the lack of combinatorial dose-response studies and assessment of their impact on MTX’s anticancer efficacy, these findings highlight their potential as adjunctive therapies for MTX-induced hepatotoxicity, warranting further research to optimize clinical applications.

## Supplementary Information

Below is the link to the electronic supplementary material.

ESM 1 Supplementary file1 (DOCX 897 KB)

## Data Availability

All data generated and analyzed during this study are contained within the published article.
