# Supplementary material for: Modulation of AMPK by esomeprazole and canagliflozin mitigates methotrexate-induced hepatotoxicity: involvement of MAPK/JNK/ERK, JAK1/STAT3, and PI3K/Akt signaling pathways
Source: Naunyn Schmiedebergs Arch Pharmacol. 2025 Mar 7;398(8):10901–19. doi: 10.1007/s00210-025-03908-3 (PMC12350602; doi:10.1007/s00210-025-03908-3)
Supplement: Supplementary file 1 — Supplementary file1 (DOCX 897 KB) [file 210_2025_3908_MOESM1_ESM.docx]

**Title:**

**Modulation of AMPK by Esomeprazole and Canagliflozin mitigates Methotrexate-induced hepatotoxicity: Involvement of MAPK/JNK/ERK, JAK1/STAT3, and PI3K/Akt signaling pathways**

**Authors & affiliations:**

Ahmed M. El-Dessouki^1^*, Mohamed E. Kaml^1^, Mohammed F. EL-Yamany^2^

^1^ Pharmacology and Toxicology Department, Faculty of Pharmacy, Ahram Canadian University (ACU), 6^th^ of October City, Giza 12566, Egypt.

^2^ Pharmacology and Toxicology Department, Faculty of Pharmacy, Cairo University, 11562, Egypt.

* **Corresponding authors:**

Ahmed M. El-Dessouki: E-mail: [ahmed.desoky@acu.edu.eg](mailto:ahmed.desoky@acu.edu.eg)

**Uncropped Western blot results included as supporting data in the main article:**

**p-JNK**


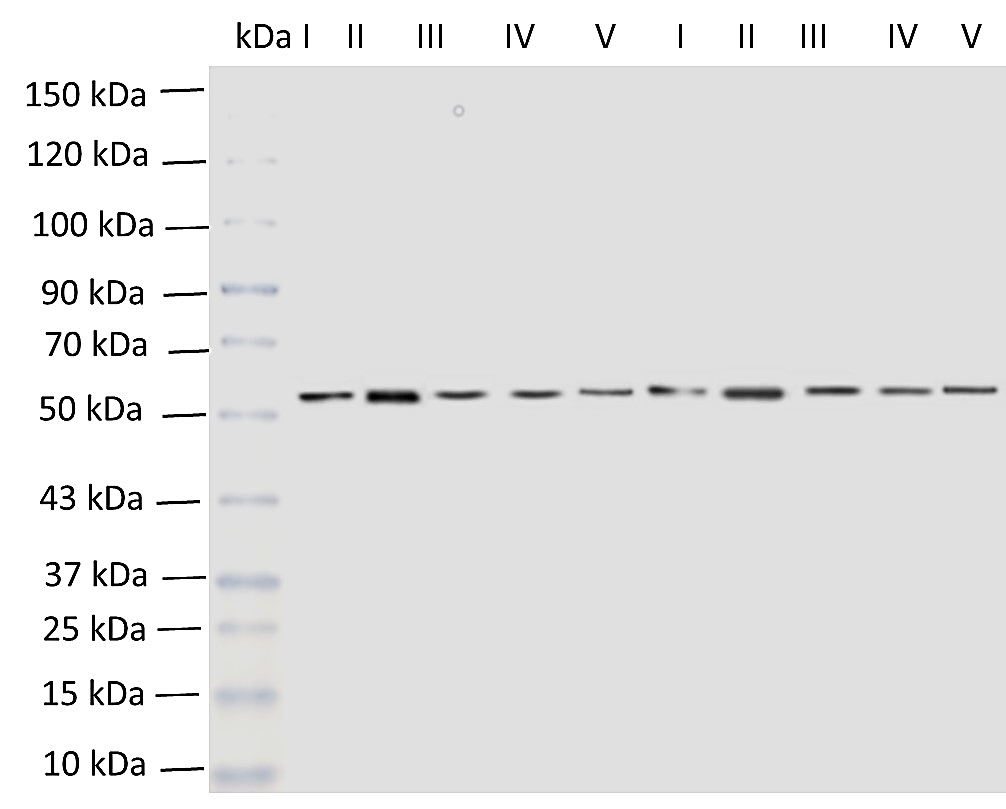


**p-ERK1**


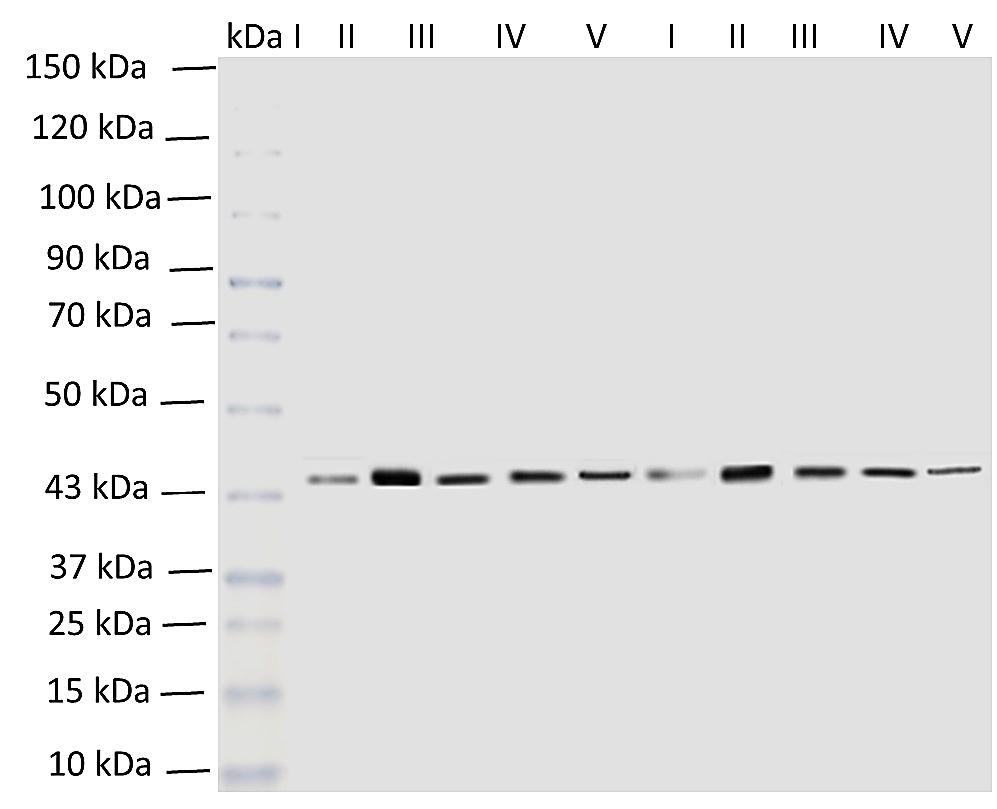


**p-p38**


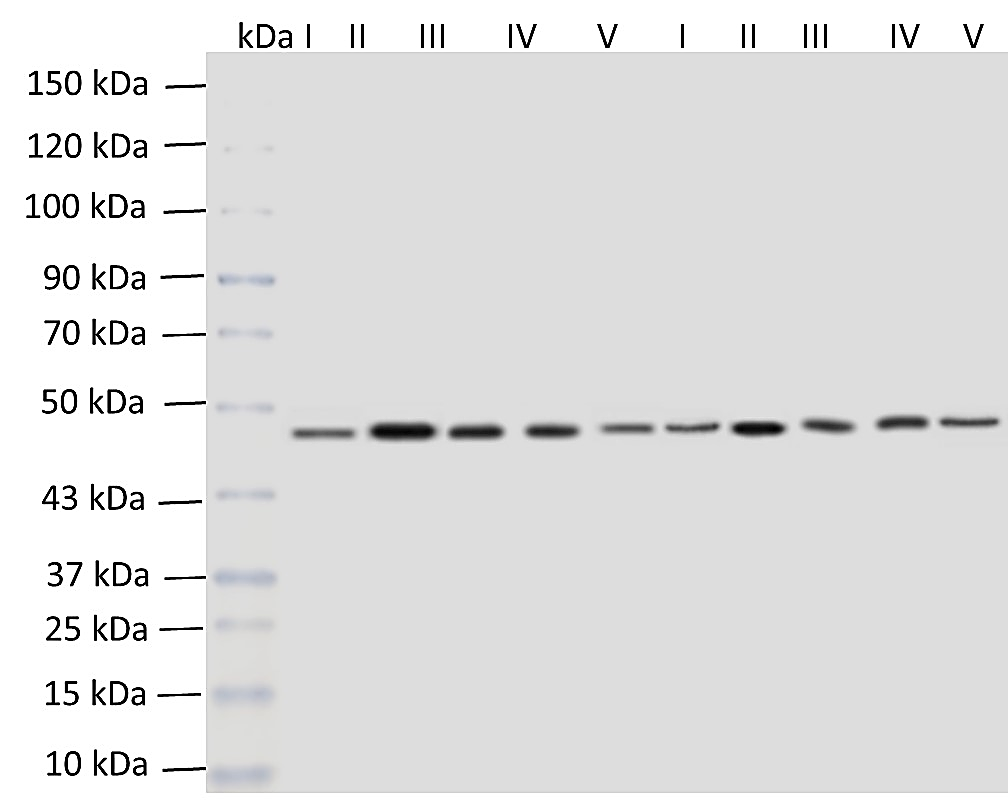


**p-JAK1**

**
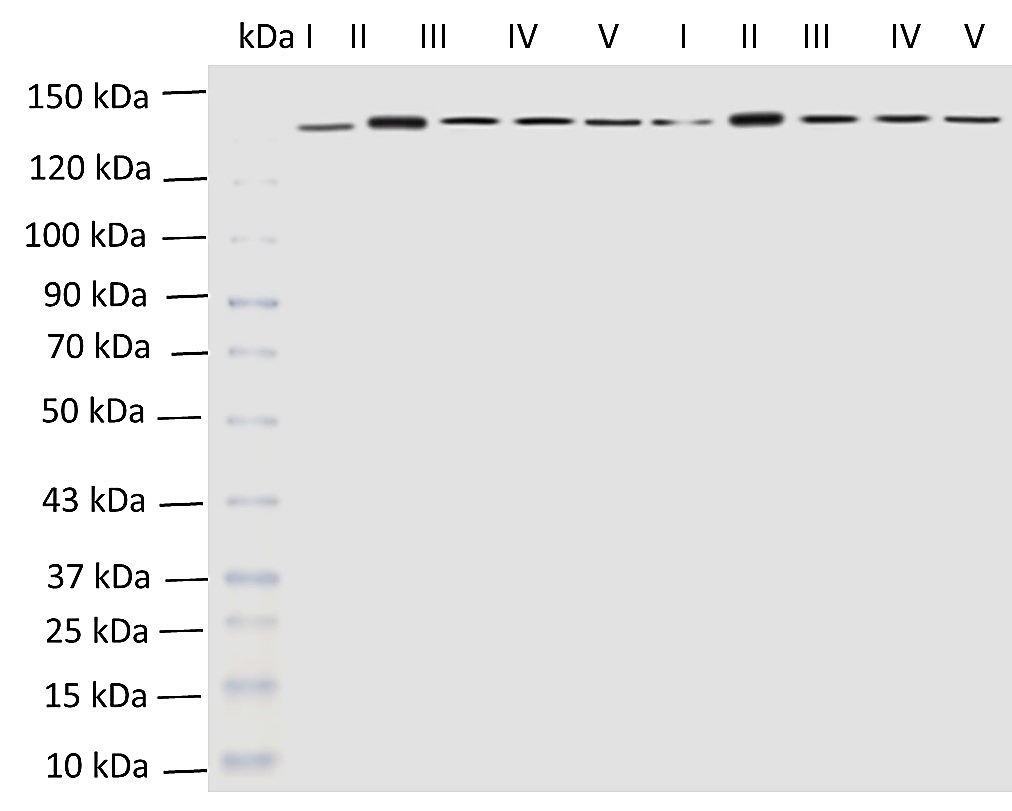
**

**p-STAT3**

**
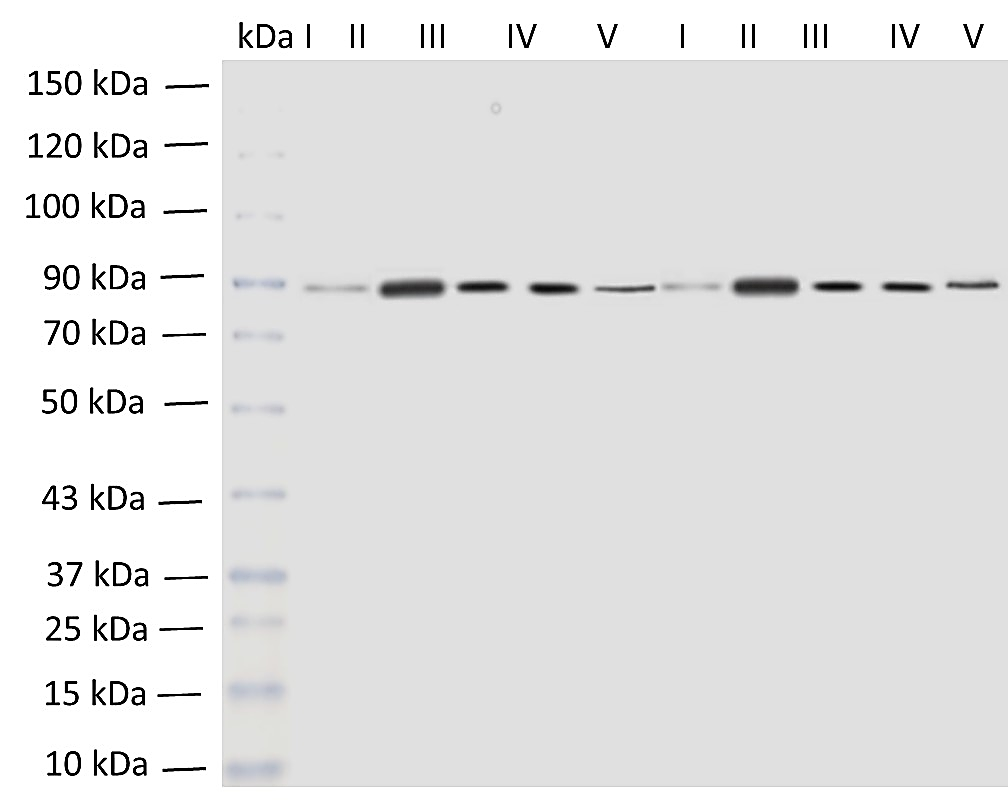
**

**β-actin**

**
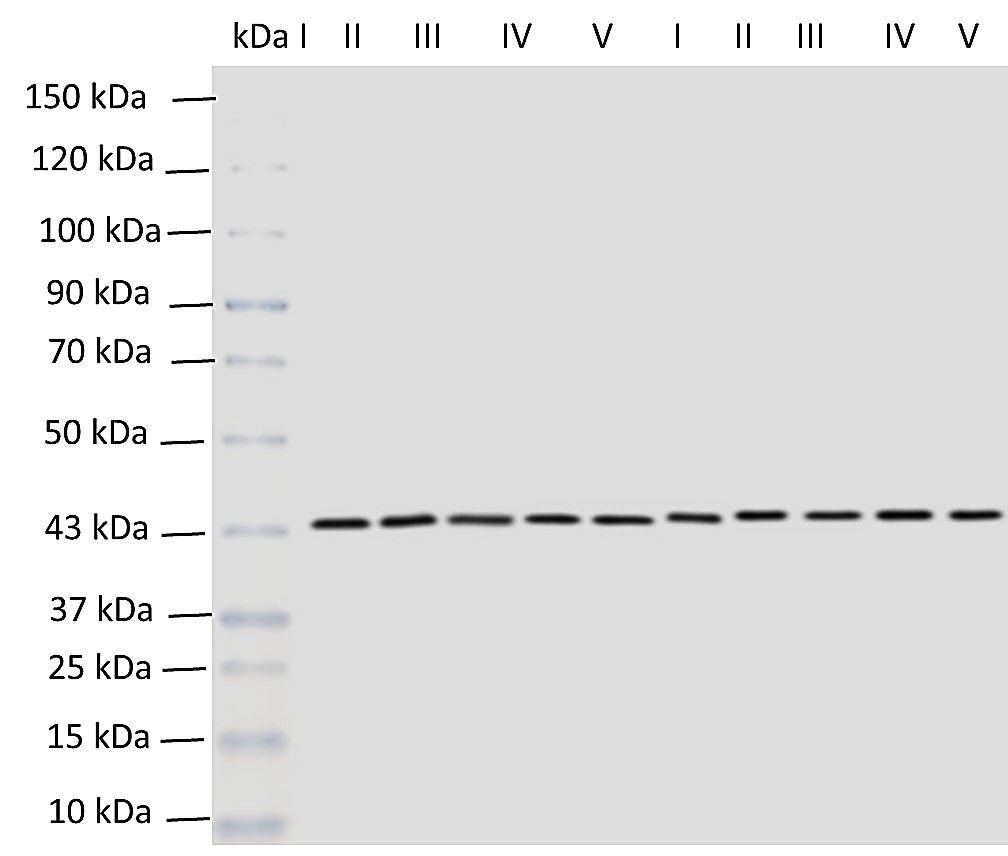
**
